# Feature-enhanced X-ray imaging using fused neural network strategy with designable metasurface

**DOI:** 10.1515/nanoph-2023-0402

**Published:** 2023-09-25

**Authors:** Hao Shi, Yuanhe Sun, Zhaofeng Liang, Shuqi Cao, Lei Zhang, Daming Zhu, Yanqing Wu, Zeying Yao, Wenqing Chen, Zhenjiang Li, Shumin Yang, Jun Zhao, Chunpeng Wang, Renzhong Tai

**Affiliations:** Shanghai Synchrotron Radiation Facility, Shanghai Advanced Research Institute, Chinese Academy of Sciences, Shanghai 201204, China; Shanghai Institute of Applied Physics, Chinese Academy of Sciences, Shanghai 201800, China; University of Chinese Academy of Sciences, Beijing 100049, China; Nanjing University, Nanjing 210093, China; Zhejiang Lab, Hangzhou 311101, China; Innovation Academy for Microsatellites, Chinese Academy of Sciences, Shanghai 201203, China

**Keywords:** X-ray optics, metasurface, convolutional autoencoder, denoising

## Abstract

Scintillation-based X-ray imaging can provide convenient visual observation of absorption contrast by standard digital cameras, which is critical in a variety of science and engineering disciplines. More efficient scintillators and electronic postprocessing derived from neural networks are usually used to improve the quality of obtained images from the perspective of optical imaging and machine vision, respectively. Here, we propose to overcome the intrinsic separation of optical transmission process and electronic calculation process, integrating the imaging and postprocessing into one fused optical–electronic convolutional autoencoder network by affixing a designable optical convolutional metasurface to the scintillator. In this way, the convolutional autoencoder was directly connected to down-conversion process, and the optical information loss and training cost can be decreased simultaneously. We demonstrate that feature-specific enhancement of incoherent images is realized, which can apply to multi-class samples without additional data precollection. Hard X-ray experimental validations reveal the enhancement of textural features and regional features achieved by adjusting the optical metasurface, indicating a signal-to-noise ratio improvement of up to 11.2 dB. We anticipate that our framework will advance the fundamental understanding of X-ray imaging and prove to be useful for number recognition and bioimaging applications.

## 1 Introduction

By converting X-rays with attenuation information into visible light images [1, 2], scintillator-based detectors enable providing valuable insights into internal structure that are of utmost importance in many fields such as healthcare diagnostics, cancer therapy, particle physics, and archeology [3–5]. Based on the photographs obtained from the detectors, doctors can make an accurate diagnosis of lung infections, and archaeologists can also examine hidden characters in ancient oil paintings thousands of years ago. In order to obtain high-quality photographs, on the one hand, a variety of scintillators with high-efficiency X-ray conversion capabilities have been evaluated, such as Ce:YAG [6], DPA-MOF [1], Tb:NaLuF_4_ [7], CsPbBr_3_ [8], and CH_3_NH_3_PbBr_3−*x*_Cl_*x*_ [9]. These novel scintillators usually possess fast activation dynamics, high X-ray sensitivity, and many other advantages, which are conducive to imaging. On the other hand, artificial neural networks (ANNs) are introduced to process digital formats captured by camera to extract features or improve signal-to-noise ratio (SNR) [10–12]. For example, in convolutional autoencoder (CAE) as a typical image processing ANN, the input is compressed into a dimensionality-reduced latent-space representation through a convolutional encoder, and then the decoder is used to reconstruct and output image. In this way, through the utilization of scintillator-based light transmission and ANN-based electronic algorithms, the information that people are interested in such as tumor tissues or nerve edges will, therefore, be highlighted in the output image, which prompts the framework to be regarded as an effective standard paradigm.

**Figure 1:**
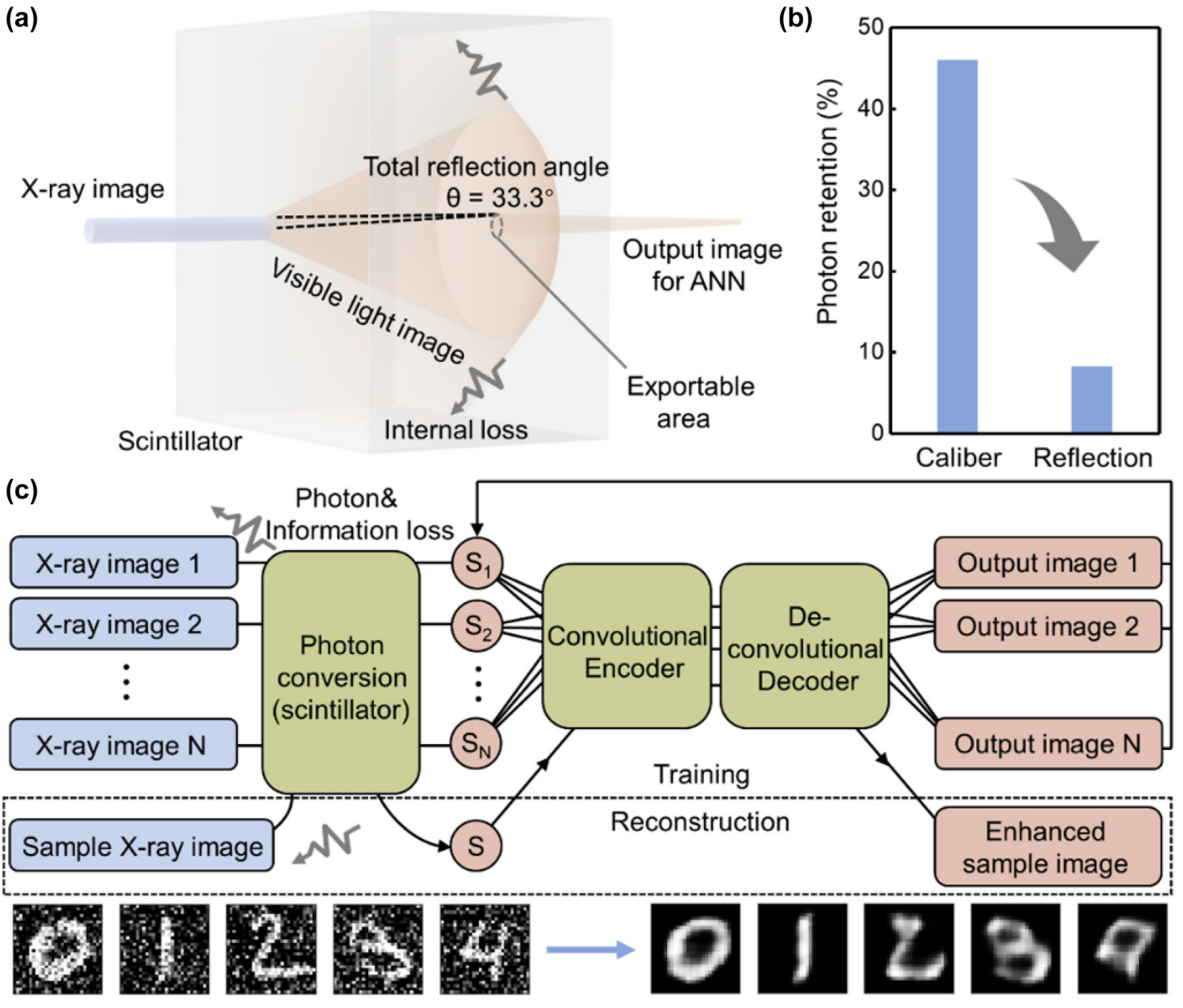
Typical separated X-ray imaging/postprocessing process. (a) The photon conversion process that occurs in the scintillator. Internal reflection caused by high refractive index. Total reflection angle is calculated based on Ce:YAG. (b) The photon retention caused by the scintillator size (caliber) and the refractive index (reflection). (c) Schematics of typical indirect X-ray imaging process with CAE postprocessing. The color of blue shows the realization in the optical process, and red shows realization in the electronic network. The dashed box shows the training process and reconstruction process. Inset: The input images contain noise (left), and the output images generated from the classic electronic CAE (right) after 800 epochs of training.

Nowadays, advanced research aims to achieve fast and high-quality reconstructed images, which are universal to all samples, implying the requirement of efficient optical image acquisition and rapid preparation of neural networks [13]. However, under the current strategy, the optical process and the electronic process are separated from each other, which lead to inherent limitations that are difficult to address. Specifically, from an optical perspective, a major problem is the notorious internal reflection caused by extremely high refractive index of scintillator, which, together with the attenuation caused by other optics, leads to massive information loss during the propagation of optical images (Figure 1a). The number of these lost photons is as high as 92 % (calculate based on Ce:YAG), which cause the degradation of images provided to ANN and the decline of SNR [14], aggravating the requirement of computing cost (Figure 1b). From the perspective of electronics calculation, the training of the neural network to the captured image is usually blind because of the ignorance of the optical process. A large amount of precollected data (Figure 1c) is necessary to train the encoder/decoder pair and to ensure well-learning for categories, which may take several hours or even several days (Supplementary Figure 1) and needs to be reconfigured when the venue or sample changes [15, 16]. Therefore, a suitable strategy for promoting the integration of optical and electrical processes is essential to help achieve the synchronous optimization of the imaging process.

Recently, metasurfaces, which constructed by global-ordered planar subwavelength microstructures with predetermined optical properties, have been proposed as an efficient optical processing element. Benefitting from the predesigned space layouts and facile nanofabrication techniques, metasurfaces have shown great promise for achieving effective control of the wavefront of light with low cost and fast speed. By imparting arbitrary spatial and spectral transformations on incident light waves, metasurfaces provide a unique ultra-high-speed optical method of implementing the computation or transformation (e.g., convolution) of the local wavefront matrix at the subwavelength scale. Therefore, taking advantage of the metasurface, here we introduce a fused neural network design strategy to integrate the optical imaging process and electronic postprocessing into a complete fused optical–electronic CAE, simply affixing an additional optical metasurface to the scintillator (Supplementary Note 1). The optical encoder of CAE is realized by imaging with a predesigned metasurface, and the electronic decoder can reconstruct the image in computer with enhanced features based on encoder after training. The predesigned metasurface as a convolutional kernel is endowed with the ability to extract features, as well as designable feature enhancement of the image can be achieved by adjusting the parameters of metasurface. As a conceptual demonstration, the experimental verification of two key feature enhancement capabilities using hard X-rays was implemented and delivered an SNR enhancement of up to 11.2 dB. Based on this strategy, the neural network will be directly connected to photon conversion process. The actual image in the scintillator that has just been converted into visible light is immediately input into CAE, avoiding the loss of optical information in the transmission. More importantly, the light propagation model can be inferred from the known physical process rather than sample images, wherefore, the decoder is able to deploy without additional training, which helps to enhance the generalization ability and providing substantial savings on computational cost [16, 17].

## 2 Design of the fused CAE system

**Figure 2:**
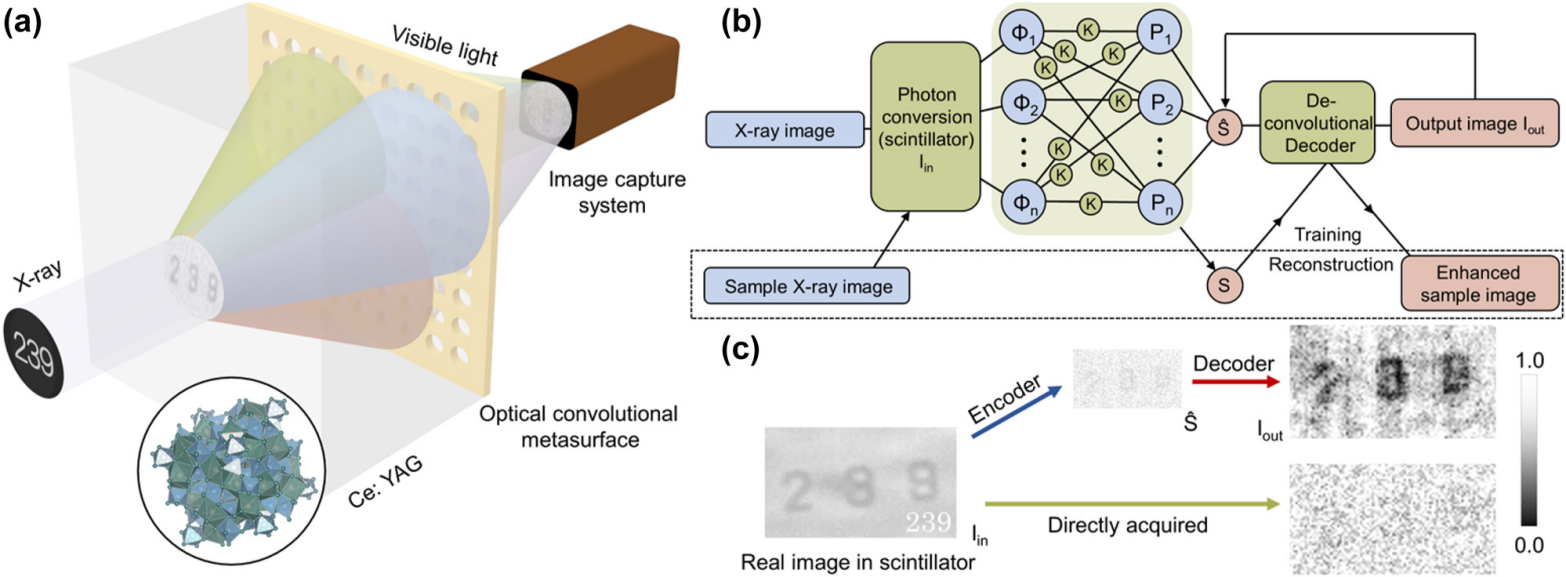
Illustration of the fused CAE. (a) Schematic illustration of the optical setup. The X-ray with image information is converted into a visible light image in the scintillator, then interacts with the optical convolutional metasurface and the resulting image is projected onto the image capture system. The color is only used to symbolize the presence of different wavefront components in the input image and does not represent the actual color of light (such as red, green, and blue) (see Supplementary Note 2 for details). (b) Schematics of the CAE framework. The color of blue indicates realization in the optical process, and red indicates realization in the electronic network. (c) Typical fused CAE results. *I*_in_ on the left is an image obtained with a long exposure time, which refers to the actual image in the scintillator and cannot actually be obtained under the same measurement time and test conditions as the images on the right. The upper route shows our proposed fused CAE, and the upper right is a typical fused CAE reconstructed image. The bottom route shows the normal X-ray imaging process and the obtained low-quality image (bottom right).

We start with the design principle of the optical encoder of fused CAE. In scintillation-based X-ray imaging process, high-energy photons of X-rays will be converted into visible light in scintillator [18] and diffracted at the exit interface (usually the scintillator–air interface). Diffraction causes most of the spatial high-frequency light to be internally reflected and only a small amount of light continues to propagate and is finally imaged on the detector; in other words, the original complete image in scintillator is convolved by a low-pass function based on the numerical aperture. From the perspective of machine learning, it is very similar to a convolutional encoder in CAE only in terms of process, and Supplementary Note 2 verifies the theoretical feasibility. Therefore, analogous to the encoder in CAE, an optical regime containing a designable optical metasurface can realize convolutional optical encoding and perform operations on the image and the convolutional kernel represented by the metasurface at the speed of light. In our design, the metasurface is fabricated by the material with a similar refractive index to scintillator (e.g., SiN_*x*_ is suitable for Ce:YAG) and is affixed to the scintillator surface away from the X-ray source (Figure 2a). It means that the metasurface will replace the simple refractive index transition surface to manipulate the wavefront Φ_*n*_ of the visible light image *I*_in_ and perform a convolutional encode operation based on the contained convolutional kernel *K* under the perspective of the neural network (Figure 2b). Without loss of generality, the optical metasurface can be predesigned in view of Huygens–Fresnel theory, allowing adjustment of physical parameters to obtain various *K* as convolutional kernels with different feature extraction capabilities, which is consistent with the encoder in CAE. Thereby, the convolutional encoder is realized optically and connected to the low-attenuation fluorescent image *I*_in_ directly, avoiding the destruction of information in optical signal chain by the internal reflection in scintillator.

We then progress to the solution formulation of the decoder. The image $\hat{S}$ captured by the detector is of lower quality because it is limited by the number of pixels of the detector; however, benefiting from the optical convolutional operation, the captured image $\hat{S}$ is composed of the convolutional images (*P*_1_, … , *P*_*n*_) after feature extraction and still contains the characteristic information of the original image *I*_in_ and can regarded as the feature vector set in CAE to be reconstructed in the decoder. It is worth noting that the training of the encoder/decoder pair in traditional CAE is specified based on the comparison of massive reconstructed images/original images of the same category. This is necessary because it is difficult to predict the feature extraction effect caused by the selected convolutional kernel, which is attributed to the fact that the convolutional kernel lacks physical meaning. Fortunately, in the fused CAE we constructed, the encoder is predesigned and prepared from diffraction theory, and the deconvolutional decoder can be thus inferred by examining the optical behavior of the encoder physical prototype. Specifically, for the real propagation process of single-channel scintillator excitation, the obtained spatial amplitude is

(1) $$P_{n}=\Phi_{n}\left[x,y;n\left(\lambda \right)\right]⊗K_{\text{des}}\left(n\right)⊗K_{\text{sys}}\left(n\right)$$

which shows the convolution operation of the wavefront function and the optical element. Here, *P*_*n*_ is the single coherent wavefront component collected in front of the detector. Φ_*n*_ is the coherent wavefront component from the image *I*_in_, where *x* and *y* are the horizontal and vertical coordinate components, *λ* is the wavelength of Φ_*n*_. ⊗ is the convolution operation, *K*_des_(*n*) and *K*_sys_(*n*) are convolutional kernel of the designed optical metasurface and other optical components in the system that response to Φ_*n*_. And the light intensity information of the incoherent pattern received on the detector is

(2) $${\left|P_{n}\right|}^{2}={\left|\underset{n\left(\lambda \right)}{\sum }\Phi_{n}\left(x,y;n\right){⊗}_{\text{des}}\left(n\right){⊗}_{\text{sys}}\left(n\right)\right|}^{2}$$

∑ is the sum symbol. When the imaging process is only regarded as incoherent imaging, the whole process should be considered as

(3) $$\hat{S}=\left[\underset{n\left(\lambda \right)}{\sum }\left|\Phi_{n}{\left(x,y;n\right)}^{2}\right|\right]⊗{\left|\underset{n\left(\lambda \right)}{\sum }K_{\text{sys}}\left(n\right)⊗K_{\text{des}}\left(n\right)\right|}^{2}$$

$\sum_{n\left(\lambda \right)}K_{\text{designed}}\left(n\right)⊗K_{\text{sys}}\left(n\right)$ Therefore, for the optical system that the encoder has determined, $\sum_{n\left(\lambda \right)}K_{\text{designed}}\left(n\right)⊗K_{\text{sys}}\left(n\right)$ is fixed, and the decoder should have the following inference target Ω; when this goal is reached, the decoder completes the learning of the aforementioned optical encoder, and a deconvolution operation can be used to reconstruct the enhanced image *I*_out_:

(4) $$\Omega ={\arg\min}_{\left(x,y\right)\in R}{\left\|\hat{S}-{\left|P_{n}\right|}^{2}\right\|}_{2}^{2}$$

which ensures the learning of decoder to the optical process, the (*x*, *y*) coordinate point should be in real space *R*. In order to demonstrate the simplified learning to the optical process rather than the image itself, we adopted a simple recurrent neural network (RNN)-derived method for training: a known sample is used to perform an imaging through the optical encoder, and RNN training is implemented to obtain the deconvolutional decoder based on the captured image as a feature vector set and the known sample image as ground truth; see Supplementary Note 3 for details. Therefore, only one imaging of a known sample is required to establish a suitable deconvolutional decoder. Nevertheless, it should be noted that the decoder training has no additional restrictions on the types of ANNs required. When both the encoder and decoder have been prepared, the fused CAE is established and applied to any category of sample. The X-ray absorption image of the sample is converted into visible light and then input to the optical convolutional encoder and further captured by the detector; the digital image is transferred to the electronic decoder for reconstruction and output (Figure 2c). It should be noted that all images presented in this study are fluorescein images captured by a visible-light charge-coupled device (CCD), corresponding to a wavelength range of 400–700 nm. Photons in the acquired wavelength range are then accumulated indiscriminately by the CCD camera into the brightness of the electronic image. The output image *I*_out_ reconstructed by the training-completed decoder can demonstrate the enhanced features and denoising effect, similar to the classic CAE network.

## 3 Texture feature enhancement CAE

### 3.1 Principle and verification

**Figure 3:**
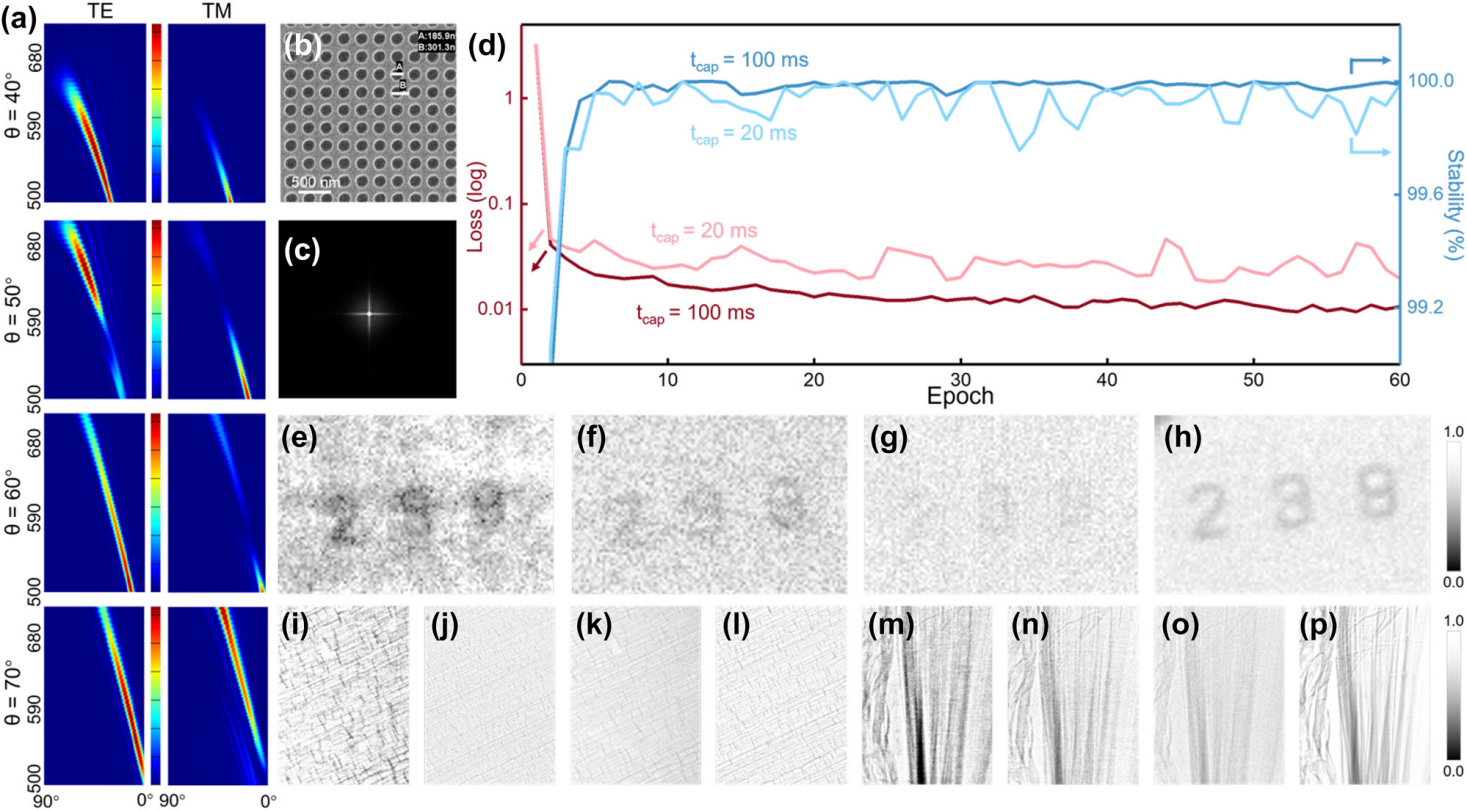
Design and training of texture enhancement convolutional kernel system (*SysK*_*ξ*3_). (a) The diffraction patterns of the metasurface with a 300 nm period for each incident angle *θ*. The longitudinal axis is the fluorescence wavelength, and the transverse axis is the exit angle (the center is 0°) for each image. The color scale is independent of each image. (b) The SEM image of the metasurface *K*_*ξ*3_. (c) Corresponding convolutional kernel of *K*_*ξ*3_. (d) Loss function and the stability during training. (e) The number “239” etched on the SiN_*x*_ film observed by *SysK*_*ξ*3_ and (f) the normal X-ray imaging system without CAE (*NSys*). (g) The feature vectors $\hat{S}$ of “239” collected by the camera. (h) Long-exposure images of “239” as a reference. The straw tissue image observed by (i) *SysK*_*ξ*3_ and (j) *NSys*. (k) The feature vectors $\hat{S}$ and (l) long-exposure images of the straw tissue as reference. The fins image observed by (m) *SysK*_*ξ*3_ and (n) *NSys*. (o) The feature vectors $\hat{S}$ and (p) long-exposure images of the fins as reference.

On the basis of the operational concept of system architecture, we propose to execute the design and implementation of actual device as preliminary proof. Texture enhancement convolutional kernel is considered beneficial to denoising and classification and first used to guide the design of the metasurface. In spatial frequency domain, the texture information of the image is contained in the high spatial frequency part of the Fourier pattern and exists as the light with a large incident angle on the exit surface of the proposed Ce:YAG [19, 20]. Consequently, light with a large incident angle needs to be transferred to center exit position by convolutional metasurface to increase the proportion of high-frequency information in the image. The finite difference time domain (FDTD) method based on Maxwell equations [21, 22] was used to simulate the metasurface to confirm the metasurface characteristics with single-order diffraction. The diffraction caused by the designed convolutional metasurface should obey the following constraints [4]:

(5) $$-1<\sin\theta <\left(\frac{1}{\xi }+\sin\varepsilon \right)/n_{\text{Ce:YAG}}$$

where *θ* is the angle at which the light in the scintillator irradiates the interface. *ξ* is the structure period of metasurface which can be adjusted. *ɛ* is the acceptance angle of the optical system, which depends on the size of the metasurface. *n*_Ce:YAG_ is the index of refraction of Ce:YAG. As shown in Figure 3a, the zero-order diffraction light disappears from the diffraction pattern with an incident angle of *θ* = 40° for the metasurface with *ξ* = 300 nm (with convolutional kernel *K*_*ξ*3_), and only the first-order diffraction light can be observed in the receiving region at even up to *θ* = 70°, covering most of the possible wavelengths of fluorescence. For the metasurface with *ξ* = 200 nm, almost no suitable light can be observed in the diffraction pattern of, implying that no information can be enhanced as shown in Supplementary Figure 2a. If the period *ξ* is increased to 400 nm, the light with a small incident angle is mainly enhanced and second-order diffraction will appear in the receiving region (Supplementary Figure 2b). Owing to the above analysis, *K*_*ξ*3_ is determined to be mapped to the metasurface to extract the textural property (Figure 3b).

Next, the X-ray imaging experiments were conducted at the BL13W1 beamline station at SSRF, where the beam specifications are as follows: energy resolution (DE/E): $<5\times 10^{-3}$ ; beam size: 45 mm(*H*) × 5 mm(*V*). We chose the X-ray radiation of 15 keV photon energy in the imaging process; therefore, the flux output is near 5 × 10^10^ ph/s/mm^2^. The scintillator with *K*_*ξ*3_ is placed in the light path to provide training data for decoder. A photon baffle located at the X-ray exit end is used to ensure that the stray light in the scintillator will not be excited outside the hole and use the proposed framework to collect intensity images (Figure 3c). Simple RNN applies to obtain the stable decoder [23], and the Euclid norm of the error matrix is used as the loss function of the training. Stability is calculated from the evolution of the absolute value of the matrix. As shown in Figure 3d, the loss and accuracy are plotted over 60 training epochs for the image with capture time of *t*_cap_ = 20 ms and 100 ms. The loss is decreasing quickly and reaches a minimum after 60 epochs. The accuracy also quickly approached and stabilized at about 100 % within a few steps. The imperfect training stability of the 20 ms image may be attributed to the small number of photons obtained. After the standard training process with step length = 60 is completed, the Richardson–Lucy deconvolution algorithm is used to carry out the back-propagation process of the convolutional metasurface as the decoder and present a complete fused optical–electronic CAE as *SysK*_*ξ*3_ [24]. We demonstrated the measured image of number “239” etched on the SiN_*x*_ film (Figure 3h) at first (Figure 3e), and the image generated by the normal X-ray imaging system without CAE (*NSys*) are also shown in Figure 3f for comparison. The images generated by our framework possess prominent “239” markings and better image quality of the edge and texture structure, which is not observed in the pictures generated by the *NSys* and the feature vectors $\hat{S}$ collected by the camera (Figure 3g), verifying the feasibility of the proposed framework. Owing to the learning of the convolutional kernel rather than the information of *I*_in_, the training-completed framework is generally suitable for all kinds of sample without modification. Therefore, the straw tissue (Figure 3l) and fins of *Danio rerio* (Figure 3p) are also employed to test the proposed neural network. As expected, consisting with the number image, the reconstructed images showed the obvious fiber structure (Figure 3i) and dark lepidotrichia (Figure 3m) compared to the $\hat{S}$ (Figure 3k and o) and the *NSys* product (Figure 3j and n), which illustrates the broad applicability of fused optical–electronic CAE in multi-category images.

### 3.2 Quantitative analysis

**Figure 4:**
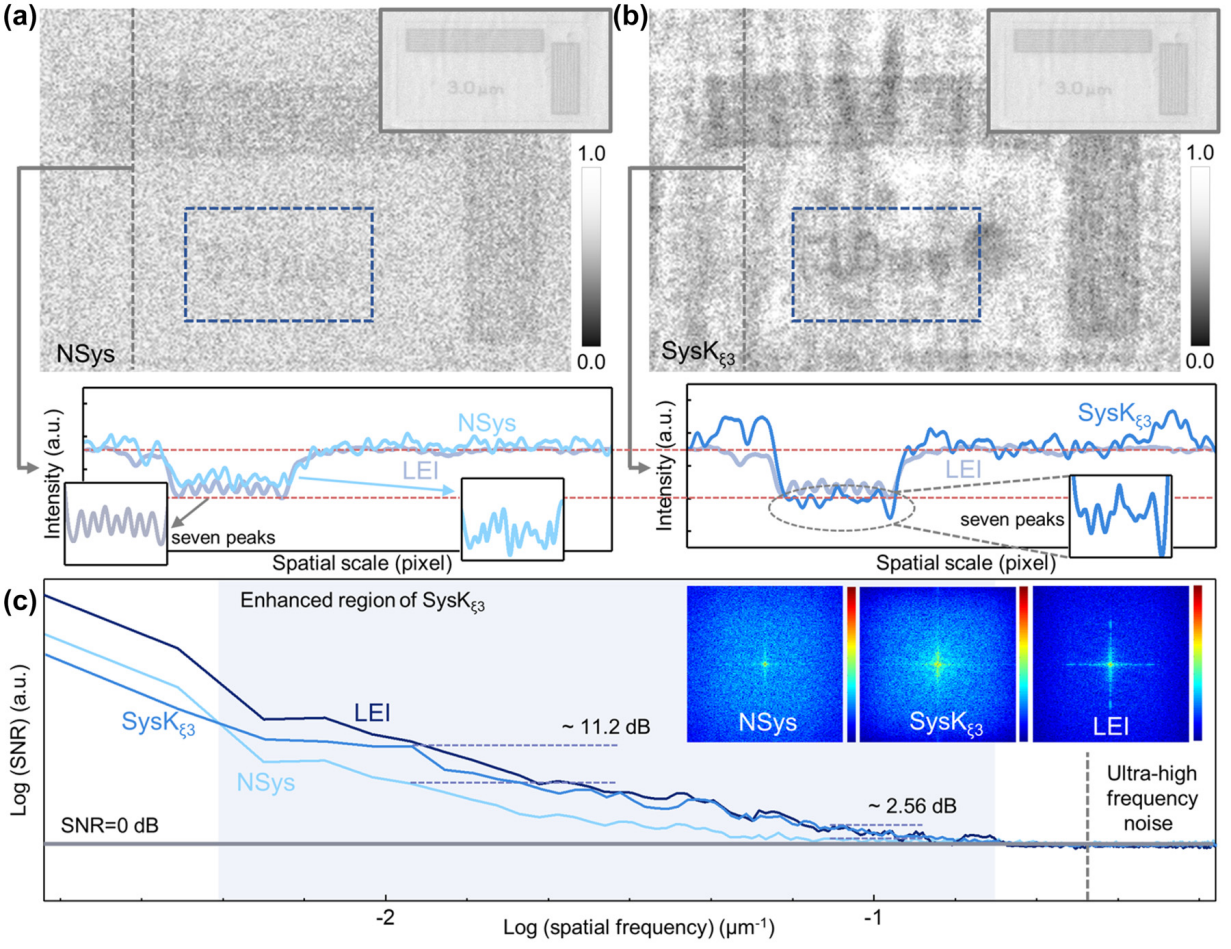
Measurement of the periodic structural sample by *SysK*_*ξ*3_. (a) The image observed by *NSys* and the corresponding strength section. Inset: LEI was used as ground truth. The detail area is specially enlarged to show the seven iconic peaks. (b) The image generated from *SysK*_*ξ*3_ and the corresponding strength section. Inset: LEI was used as ground truth. The detail area is specially enlarged to show the seven iconic peaks. (c) The curves of SNR in the whole spatial frequency range. The light blue area indicates the enhanced spatial frequency region. The 2D Fourier transform spectra of *NSys*, *SysK*_*ξ*3_, and *SysK*_*ξ*5_ are shown in inset.

Furthermore, we analyzed the feature extraction properties of *SysK*_*ξ*3_ quantitatively. The response of fringes with fixed 3 μm spacing was measured. The intensity ratio of the stripe region to the blank region is further calculated to be 0.91 and 0.804 for the original image (Figure 4a) and the image generated by *SysK*_*ξ*3_ (Figure 4b), respectively. Considering that the proposed sample is imaged by absorption, a lower intensity ratio indicates more obvious enhancement and can be further calculated as a 1.14 times contrast-to-noise ratio (CNR) improvement in the enhanced area. In addition, the characteristics of “3 μm” are highlighted in Figure 4b, and the seven intensity peaks corresponding to seven bright lines in the region can be also distinguished, but these characteristics cannot be observed in *NSys*. The compressed $\hat{S}$ is shown in Supplementary Figure 3. The Fourier transform spectrum was further obtained, which indicates the enhancement of the orderly high-frequency signal (Figure 4c) and consistent with the above analysis. Therefore, it is necessary to calculate the SNR in the full frequency domain to provide direct analysis, and long-term exposure image (capture time > 100 ms, LEI) was used as ground truth (Figure 4a and b). Owing to the need for sampling accuracy, SNR data were collected from the corresponding large-area images (Supplementary Figure 4) and normalized based on ultra-high frequency noise. As shown in Figure 4c, the image generated by *SysK*_*ξ*3_ provides an excellent SNR as high as 27.73 dB, which is approximately 11.2 dB higher than that of the original image and very close to the SNR of the LEI of 29.36 dB at the same spatial frequency (∼10^−2^ μm^−1^). Even in the ultrahigh-frequency band (∼10^−1^ μm^−1^), the image generated by *SysK*_*ξ*3_ still indicates an enhanced SNR, which is 2.56 dB higher than the original image, similar to the LEI (∼3.52 dB). The increase in high-frequency components in the spatial spectrum analysis can be observed as expected, which demonstrates the texture features enhancement capabilities of the proposed *SysK*_*ξ*3_.

As a comparison, the decoder trained by the same RNN process was also applied to the *NSys* and measured to exclude the influence of the deconvolution algorithm on the generator. In this situation, except for the metasurface, the framework is consistent with the proposed fused CAE. As shown in Supplementary Figure 5, the image generated in the *NSys* with decoder by the same algorithm does not possess similar feature enhancement to that *SysK*_*ξ*3_, which implies that the denoising effect of the Richardson–Lucy algorithm on the image is limited, and the enhanced high-frequency contribution is considered to come from the eigenvector set extracted from the input dataset. Ordinary image denoising algorithms such as the Gaussian filter [25], mean filter [26], median filter [27], low-pass filter [28], and two kinds of wavelet filters [29] were also measured. As shown in Supplementary Figure 6, the feature enhancement is not obvious compared with the image generated by the proposed framework. This can be attributed to the selection of convolutional kernels, and the bias toward light with a large incidence angle enhances the texture rather than the entire image.

### 3.3 Synchrotron X-ray tomography

**Figure 5:**
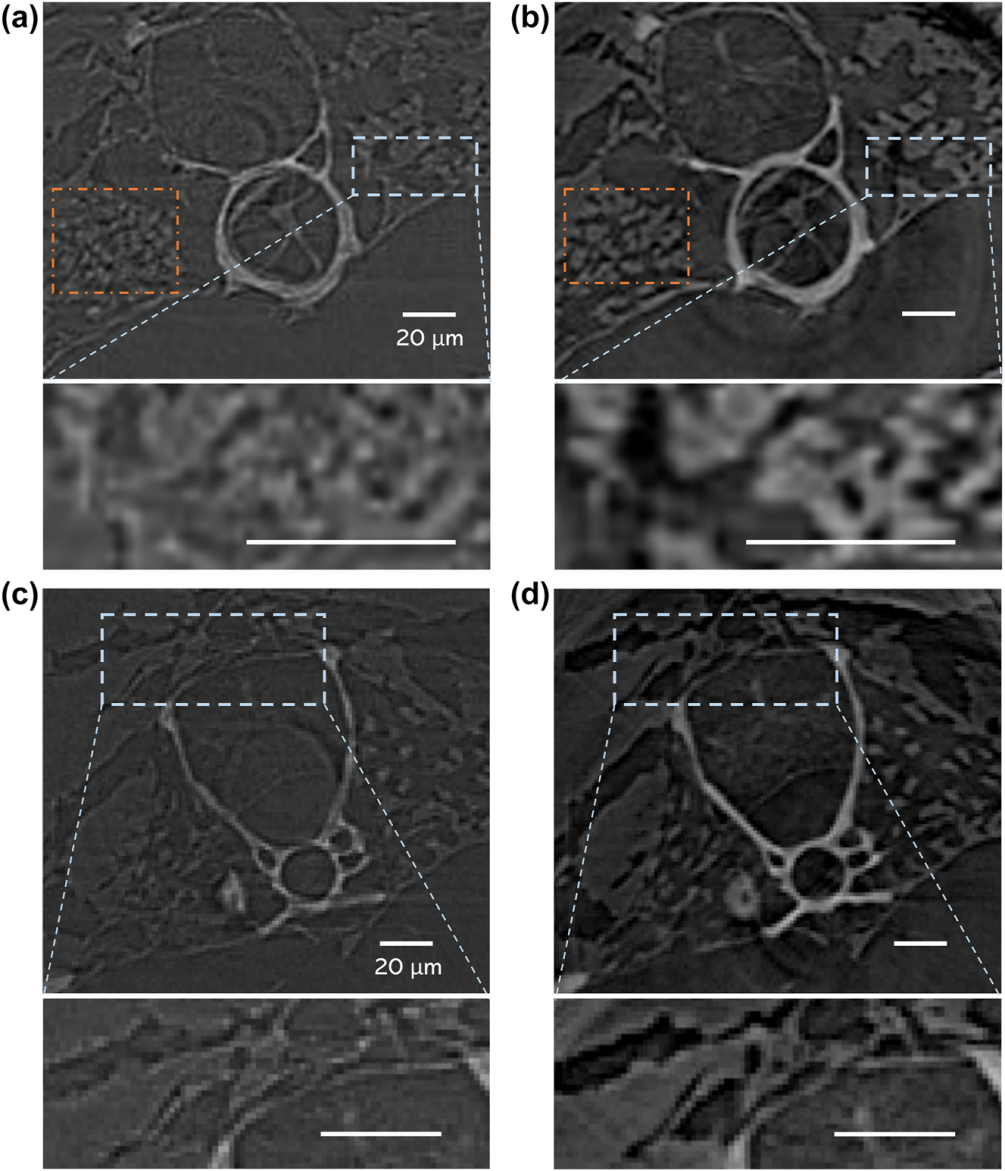
Tomography results for different X-ray imaging frameworks. Reconstructed slices and the enlarged view of the select area of the vertebra via (a) *Nsys* and (b) *SysK*_*ξ*3_. Reconstructed slices and the enlarged view of the select area of the rib via (c) *Nsys* and (d) *SysK*_*ξ*3_. The brightness of background noise is adjusted to the same level.

As a practical exploration of the proposed optical–electronic CAE applied to X-ray imaging, a tomography experiment was conducted to verify and demonstrate its technical applicability. The experiment was carried out at the BL13W1 beamline station at SSRF, where 900 projection images of a biological sample were obtained at different angles using the *SysK*_*ξ*3_ and *NSys* imaging frameworks, respectively. The exposure time was set to 100 ms and the Gridrec reconstruction algorithm [30] was used to realize the slice reconstruction from the projections. The zebrafish *D. rerio* was used as the biological sample, of which the reconstructed vertebra and rib slices are shown in Figure 5. As can be seen from the figure, the reconstructed slice is of extremely high quality as *SysK*_*ξ*3_ significantly increases the SNR and CNR of each projection. Compared with the conventional X-ray imaging system without CAE (*NSys*, Figure 5a and c), the reconstructed slices utilizing *SysK*_*ξ*3_ (Figure 5b and d) reveal clearer tissue contours and sharper skeleton edges. The enlarged details of the selected areas deeply demonstrate that the proposed framework provides higher quality images with better edge and texture features, thus achieving high-fidelity reconstruction. The calculations using the perception-based image quality evaluator (PIQE) and natural image quality assessment method (NIQE) corroborated this improvement in image quality (Supplementary Note 6) [31, 32]. Moreover, as a contribution of 900 projections achieved by *SysK*_*ξ*3_, the reconstructed slice indicates that *SysK*_*ξ*3_ enhances the common features of the sample from different angles, rather than randomly generating artifact signals, confirming the potential of the proposed framework in practical application.

## 4 Regulation of feature enhancement trends

**Figure 6:**
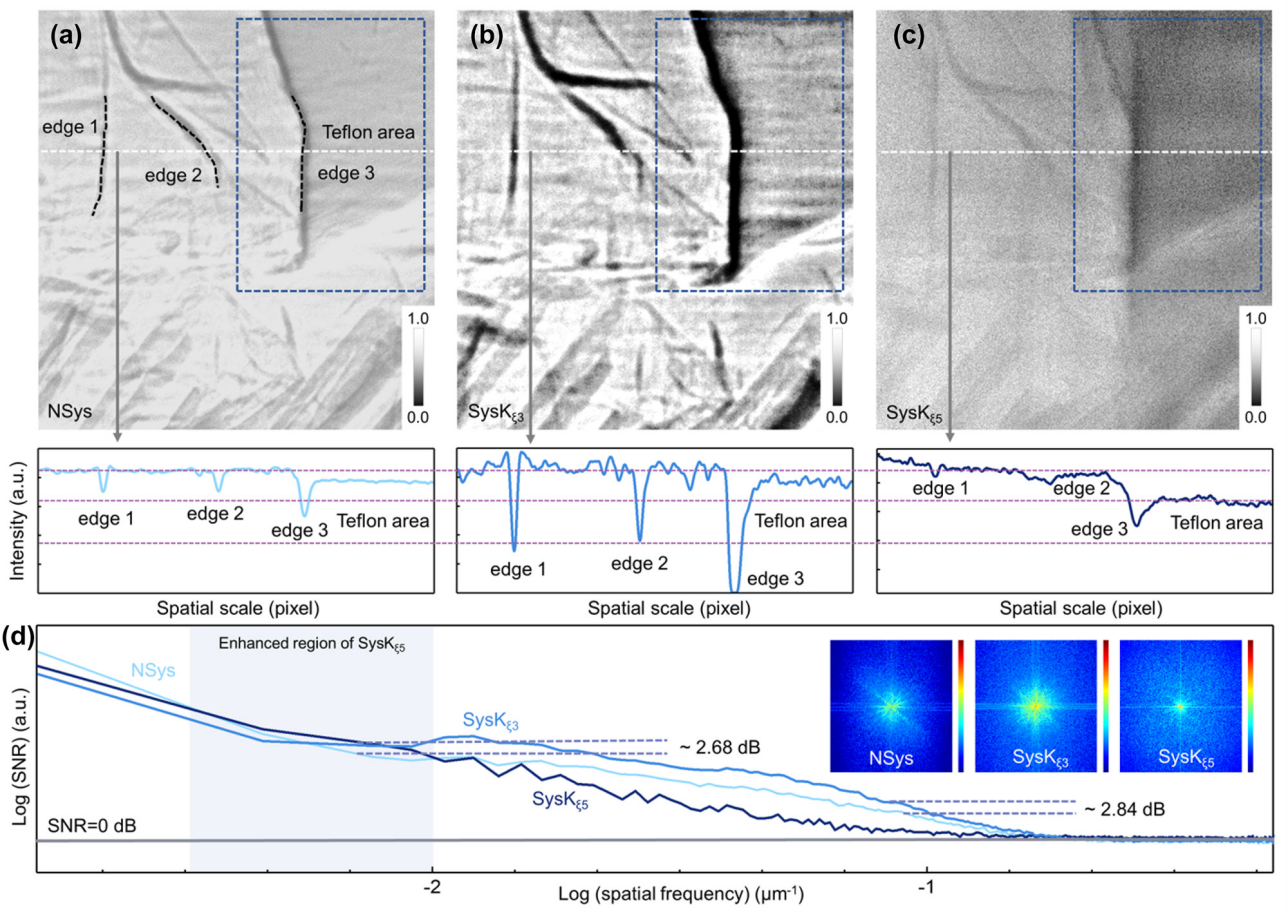
Measurement of proposed framework with multiclass convolutional kernels. (a) Image of the fin placed in a Teflon tube observed by *NSys*, (b) *SysK*_*ξ*3_, and (c) *SysK*_*ξ*5_ with the corresponding strength section. The blue box indicates the contrast between the two different enhancement features. (d) The curves of SNR in the whole spatial frequency range. The light blue area indicates the enhanced spatial frequency region. The 2D Fourier transform spectra of *NSys*, *SysK*_*ξ*3_, and *SysK*_*ξ*5_ are shown in inset.

**Figure 7:**
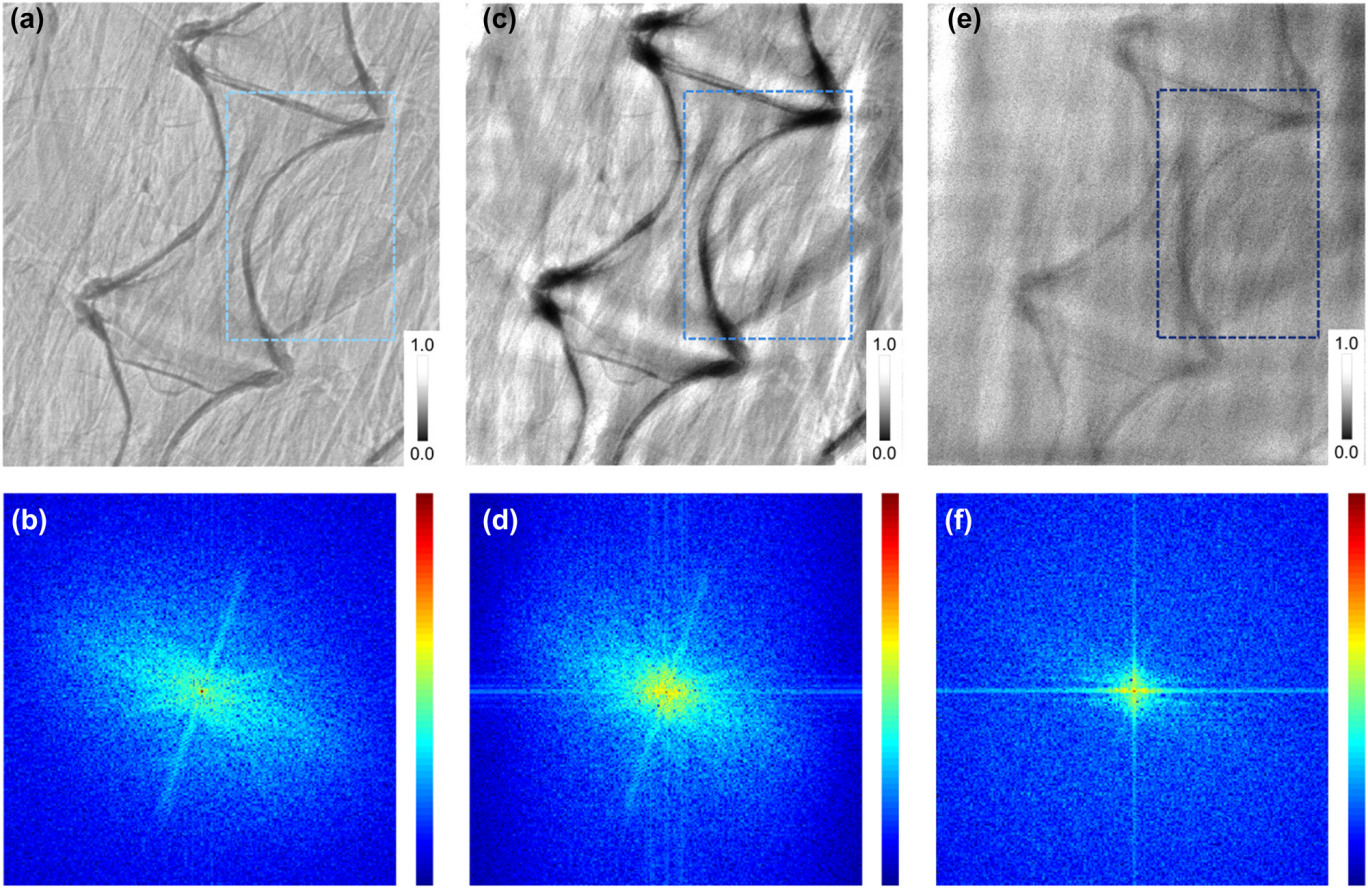
Spine images observed by different frameworks. (a) The spine image observed by a camera system equipped with a scintillator (direct imaging) and (b) the corresponding 2D Fourier transform spectrum, (c) by *K*_*ξ*3_ and (d) the corresponding 2D Fourier transform spectrum, and (e) by *K*_*ξ*5_ and (f) the corresponding 2D Fourier transform spectrum. The blue box indicates the contrast between the two different enhancement features.

To further verify the extension and practical application ability of the proposed framework, convolutional kernels of regional feature enhancement are also manufactured to provide various feature extraction capabilities. The isotropic periodic optical structure with *ξ* = 500 nm (with convolutional kernel *K*_*ξ*5_) was fabricated as shown in Supplementary Figure 7, and corresponding simulation indicates that the increased photons are concentrated in the low-frequency region (Supplementary Figure 8), which drives the collective enhancement of adjacent areas, i.e., the enhancement of regional feature. The fin edge (Figure 6a) and spine (Figure 7a and b) of *D. rerio* are used to test the proposed fused CAE with *K*_*ξ*5_ (*SysK*_*ξ*5_). The fin was placed in a Teflon tube to compare different absorption coefficient images. Therefore, in contrast to the enhancement of the region edge (Figure 6b) and spine edge (Figure 7c and d) by *SysK*_*ξ*3_, the absorption difference between the material and the muscle region is clearly distinguished by *SysK*_*ξ*5_, as shown in Figures 6c and 7e and f. The corresponding intensity section reveals the special enhancement trend of regional feature. The depression on the far right of the curve is considered the Teflon region, and it can be observed that the depression in Figure 6c is more obvious and flatter than those in Figure 6a and b.

Further calculation shows that the intensity ratio of the texture region represented by the fin to the selected blank region is 0.881, 0.641, and 0.694 in Figure 6a–c, respectively. For the region of Teflon, the intensity ratio of Teflon to the blank area is calculated to be 0.858, 0.756, and 0.532, respectively, showing different enhancement characteristics. The three deep valleys on the left are attributed to the two textures and the edge of the Teflon region. Therefore, the texture features enhanced by *SysK*_*ξ*3_ shows obvious edges in Figure 6b, while the deep valleys in Figure 6c are weakened, indicating different enhancement tendencies. In addition, as a comparison of imaging enhancement effects, Laplacian sharpening and low-pass operation are also shown in Supplementary Figure 9. As more complete optical information is obtained, the system exhibits a more excellent enhancement effect as expected.

Moreover, SNR (Figure 6d) and CNR data are also collected from the corresponding images. The image generated by *SysK*_*ξ*5_ shows an enhanced low-frequency (∼7 × 10^−3^ μm^−1^) SNR as high as 32.93 dB, which is 2.68 dB greater than that in the original image, but the signal mapped by texture features is even weaker than that in the original image, as shown in the high-frequency region. Correspondingly, the image generated by *SysK*_*ξ*3_ shows that the high-frequency SNR is as high as 9.32 dB, as expected, and that the SNR of the original image in the same position (∼10^−1^ μm^−1^) is only 6.48 dB, indicating the contrast between different enhancement regions. The CNR comparison based on the Teflon material area and fin area also shows a reverse trend for the different convolutional kernels used. For the original image, the CNR of the Teflon area and fin area was calculated to be 1.030, close to 1, while the image generated by *SysK*_*ξ*3_ indicated a lower CNR of 0.849 owing to the enhancement of the area with obvious texture. However, the image generated by *SysK*_*ξ*5_ indicated a CNR as high as 1.304, far beyond the original CNR, indicating the inversion caused by enhancement of the regional feature. Therefore, the regional feature enhancement characteristics of *SysK*_*ξ*5_ are confirmed by the above results. In addition, the corresponding frequency-domain patterns are displayed in Figure 6d, which further reveals the different enhancement trends of the low-frequency region and the high-frequency region attributed to feature enhancement abilities of different convolutional kernels.

## 5 Discussion and conclusion

Therefore, we have developed a fused optical–electronic CAE applied to X-ray imaging by synergistically combining optical metasurfaces and neural network methods. Our framework allows for the generation of feature enhanced images directly from fluorescent images gained in scintillator and has been successfully validated by implementing two representative types of application by adjusting the structure of the metasurface in the experiment. Compared with the unprocessed image, the reconstructed image has distinct texture features (or regional features) and improves SNR. In addition, benefiting from the learning to the imaging process instead of the input image, the same device can be employed for various purposes immediately, without precollecting large amounts of data for a certain type of sample [16]. These characteristics greatly broaden the utilization scenarios and potential usefulness of the proposed framework.

In our current implementation, the design using diffraction equations can only produce simple periodic structures. It is still possible to manufacture complex structured metasurfaces, such as octagon or L-shaped (Supplementary Figure 10), although the effect cannot be accurately known in advance (Supplementary Figure 11). By breaking the structural symmetry of the metasurface and using higher design degrees of freedom, such as a chiral metasurface with L-shape elements, one can explore the asymmetric transmission of image information in different polarization modes, which is an effective approach for further expanding the design of convolutional kernels [33, 34]. Successive fine control of the optical field information can be achieved through the expansion of the structural parameter space. From the perspective of development prospects, further expansion of convolutional kernel requires the concept of diffractive neural network to design, that is, the network can be trained off-line using computer simulations, and the predetermined photoresponsivity matrix is then mapped to the metasurface [35, 36]. In the meantime, more training time and algorithm design are required, which prolongs the preparation time. However, compared to conventional numerical ANNs, proposed fused CAE still has a very low demand for precollected data and possesses advantages. As a typical bottleneck in conventional numerical ANNs, the massive demand for training data is difficult to achieve in practical applications, especially for synchrotron X-ray imaging. The main reason is that the uncertainty of the site and time will directly affect the photon flux and stray noise, and the scarce synchrotron radiation resources do not allow long-term data collection. Under these constraints, conventional numerical ANNs can only be trained blindly like “taking a chance,” making it impossible to compare with our method in the same scenario. Therefore, we believe that the proposed fused optical–electronic CAE has unique application potential in synchrotron radiation sources.

Another issue worth discussing is that although neural networks with optical physical designs do not offer flexibility comparable to numerical ANNs, they still have advantages in computational speed due to the unparalleled speed of light, low power consumption, and potential parallel computing power [37]. Although it is difficult to compare optical neural networks and numerical ANNs for speed and energy consumption due to different physical design and connection latency, a reliable number is that optical neural network can achieve ∼6 times the speed of GPUs (NVIDIA-TitanX) for 4-megapixel images, and it is further enhanced as the number of pixels increases because the optical calculation speed is independent of the pixel number [38]. Such high-speed computation and huge data throughput allow optical neural networks to overcome some of the unrealistic computational demands faced by numerical ANNs; therefore, this kind of neural networks can be particularly used for those involving huge computational load and on-the-fly data processing, such as real-time reconstruction of living images and intraoperative X-ray image processing [36]. A typical example, as we describe, is the CAE processing of synchrotron beamline X-ray imaging, because of the unavoidable complex convolution and deconvolution processes therein: if we convolve an input image containing *m* × *m* number of pixels with a kernel of shape, *n × n* yields a computational complexity as high as $O\left(m^{2}n^{2}\right)$ . The amount of data processing caused by convolution operations on large images is prohibitive let alone achieves real-time processing; thus, it is very necessary to apply optical neural networks to solve this difficult problem. We also realized that, compared to the almost unlimited calculation speed of light, the decoding calculation on the computer weakens the image processing speed in the proposed framework. Considering that the image sensor itself can be trained as an artificial neural network, we suggest that replacing the detector with a programmable distributed vision sensor is expected to lift the restriction on computing speed toward ultrafast CAE in further research [39, 40].

In conclusion, the neural network with designable optical convolutional metasurface proposed here has been successfully used to enhance the features of X-ray images. We anticipate that the proposed approach will accelerate the development of imaging modalities as critical support for fast and accurate modern X-ray imaging technology and provide various possibilities for dynamic imaging.

## Supplementary Material

Supplementary Material Details
