# Supplementary material for: Feature-enhanced X-ray imaging using fused neural network strategy with designable metasurface
Source: Nanophotonics. 2023 Sep 25;12(19):3793–805. doi: 10.1515/nanoph-2023-0402 (PMC11635955; doi:10.1515/nanoph-2023-0402)
Supplement: Supplementary file 1 — Supplementary Material Details [file j_nanoph-2023-0402_suppl_001.docx]

Supplementary information

**Feature-enhanced X-ray imaging using** **fused neural network strategy with designable optical metasurface**

**Hao Shi,^1,2,5^ Yuanhe Sun,^1,2,5,*^ Zhaofeng Liang,^2^ Shuqi Cao,^3^ Lei Zhang,^4^ Yanqing Wu,^1,2,*^ Daming Zhu, ^2^ Zeying Yao, ^1,2,5^ WenQing Chen, ^6^ Zhenjiang Li, ^1,2,5^ Shumin Yang, ^1,2^ Jun Zhao, ^1,2^ Chunpeng Wang, ^2^ and Renzhong Tai^1,2,*^**

^1^ Shanghai Synchrotron Radiation Facility, Shanghai Advanced Research Institute, Chinese Academy of Sciences, Shanghai 201204, China

^2^ Shanghai Institute of Applied Physics, Chinese Academy of Sciences, Shanghai 201800, China

^3^ Nanjing University, Nanjing 210093, China

*^4^* *Zhejiang Lab, Hangzhou 311101, China*

^5^ University of Chinese Academy of Sciences, Beijing 100049, China

^6^ Innovation Academy for Microsatellites, Chinese Academy of Sciences, Shanghai 201203, China

*^*^* *sunyuanhe@sari.ac.cn, wuyanqing@sinap.ac.cn, tairz@sari.ac.cn*

**Supplementary Note 1：Fabrication of metasurface**

A two-dimensional optical metasurface with a periodicity of 300nm and a filling factor of 60% was fabricated using electron beam lithography (EBL) and inductively coupled plasma (ICP) etching. All the optical metasurfaces are composed of SiN_x_ layers on the scintillator. Supplementary Note Fig. 1 illustrates the fabrication process of the encoder. Firstly, a 150nm-thick silicon nitride (SiN_x_) film was deposited on a 200mm-thick Ce: YAG scintillator thin film using chemical vapor deposition (Oerlikon PECVD 790+). Prior to this, it is necessary to pre-evaporate the indium tin oxide layer, considering the subsequent EBL process. Then, a 180nm-thick layer of PMMA was spin-coated on top of the SiN_x_ film, followed by the deposition of a 5nm-thick chromium (Cr) layer by thermal evaporation. Afterward, the sample was immersed in the solution prepared with 1.5g of Cerium (IV) Ammonium Nitrate (CAN, with the chemical formula (NH₄)₂Ce(NO₃)₆), 1.5g of concentrated nitric acid (65%), and 7g of deionized water for 10s to remove the chromium layer. The scintillator film was then rinsed with deionized water and dried with nitrogen gas. Next, the PMMA layer was soaked in a MIBK:IPA (1:3) photoresist solution for 45s, resulting in the formation of a two-dimensional array pattern on the PMMA layer. The two-dimensional periodic pattern was etched into the SiN_x_ layer using ICP etching (Oxford Plasmalab System100 ICP 180), creating a two-dimensional high-density grating. Finally, the sample was ultrasonically cleaned for 10 minutes in a mixture of acetone, ethanol, and deionized water to remove the PMMA layer.


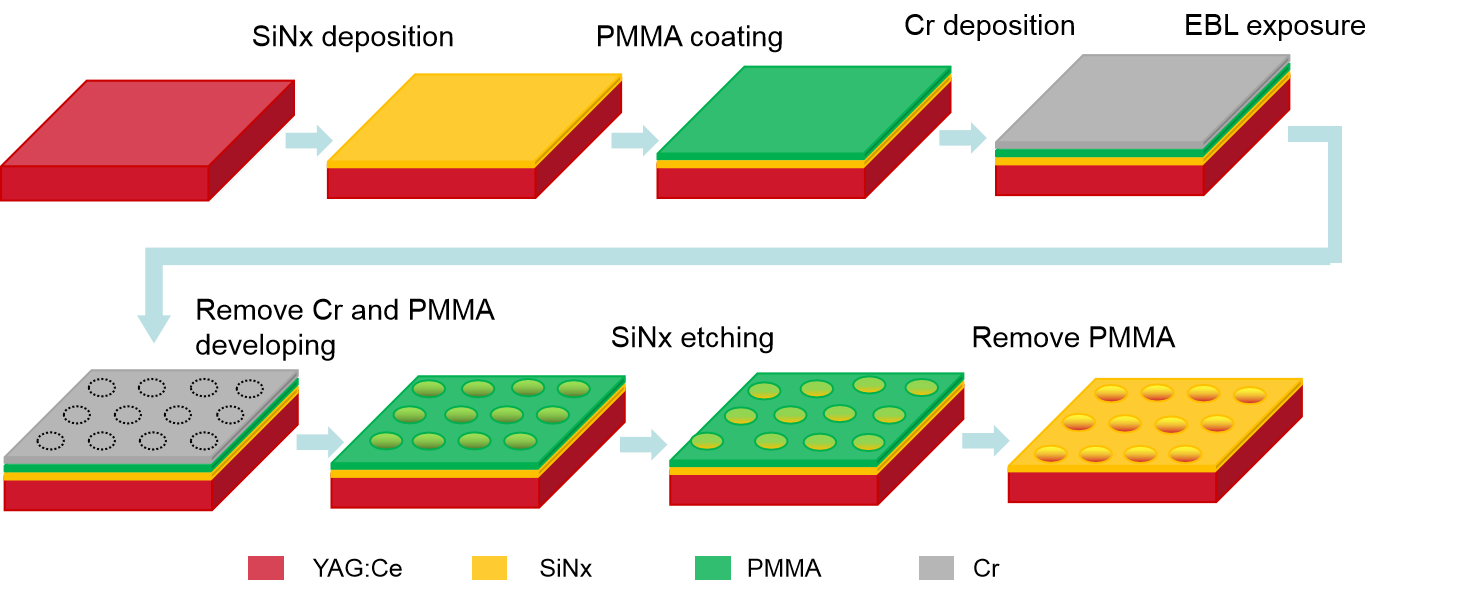


Supplementary Note Fig. 1. Diagram illustrating the fabrication process of the encoder

The resulting optical encoder had a periodicity of 300nm, an area of 1mm×1mm, a hole diameter of 180nm (60% filling factor), and an etch depth of 150nm. The scintillator Ce: YAG used in the experiment was purchased from Crytur, Czech Republic, with a sample diameter of 25.4×Dep.0.2mm. It was double-side polished with a refractive index of 1.82 and a central emission wavelength of 550nm.

**Supplementary Note 2：The theoretical feasibility of fused CAE encoder**

As a typical generation model based on convolutional neural networks, convolutional autoencoders (CAEs) have been widely studied in recent years because of their excellent performance in image compression and noise reduction. Similar to the general autoencoder, it uses convolutional layers as encoder instead of variational inference encoders to extract the features of the input dataset and uses a specified generator to reproduce it from the compressed eigenvector set. Therefore, we can replace the complex convolution steps by constructing optical convolutional layers.

In our proof-of-principle experiment, the carefully designed SiN_x_ optical metasurface was added on the back surface of Ce: YAG scintillator to realizes direct operation of X-ray fluorescence images. As original X-ray image, the input image (I_in_) was excited by X-ray in Ce: YAG scintillators (thickness 200 μm) with the absorption intensity information of samples, which is based on the luminescence mechanism of the 5d-4f radiative transitions in cerium atoms at room temperature. Due to the characteristics of indirect scintillators, disordered phonons from the crystal structure participate in the fluorescence emission process and incoherent images generated in thin Ce: YAG. When we carefully examine the process of photon conversion, it should be noted that the declared incoherent image I_in_ is actually composed “incoherently” of coherent images Φ_n_, *i.e.*, a plurality of simply arranged coherent image input nodes, and the image composed of coherent light at time *T* is emitted by some but not all cerium atoms and possesses similar wavelengths (λ ~ λ+Δλ), initial phases, and propagation directions. For the sake of simplicity, we only do further analysis on different wavelengths; other factors should have little effect on the design of the system. Considering the fluorescence emission process involving completely disordered phonons, the input image is reconstructed into an Φ_n_ set (Φ_1_,…, Φ_n_) with additional information. The board emission spectrum of Ce: YAG (FWHM~120 nm) and homogeneous doping of cerium atoms (Supplementary Note Fig. 2) guarantee the number and uniformity of Φ_n_ set. Therefore, these images remain basically similar, and each image can reflect the overall condition of I_in_.

As stated above, the proposed incoherent image I_in_ can be separated into a superposition of the coherent image Φ_n_, and interact with the metasurface separately to ensure the implementation of the convolution. However, since the decay time of Ce: YAG is much greater than that of the detector and the distribution of cerium atoms far exceeds the detector pixel density, although these images interact with the convolutional metasurface in the form of coherent light and obey the Abbe principle of image formation, we can ultimately obtain only the superimposed incoherent image on the detector. Obviously, the incoherent image Ŝ captured by the detector is greatly degraded compared to the “original image” I_in_ in the scintillator; therefore, it is not advisable to start CAE processing from the captured image Ŝ compared to the proposed method starting from I_in_.


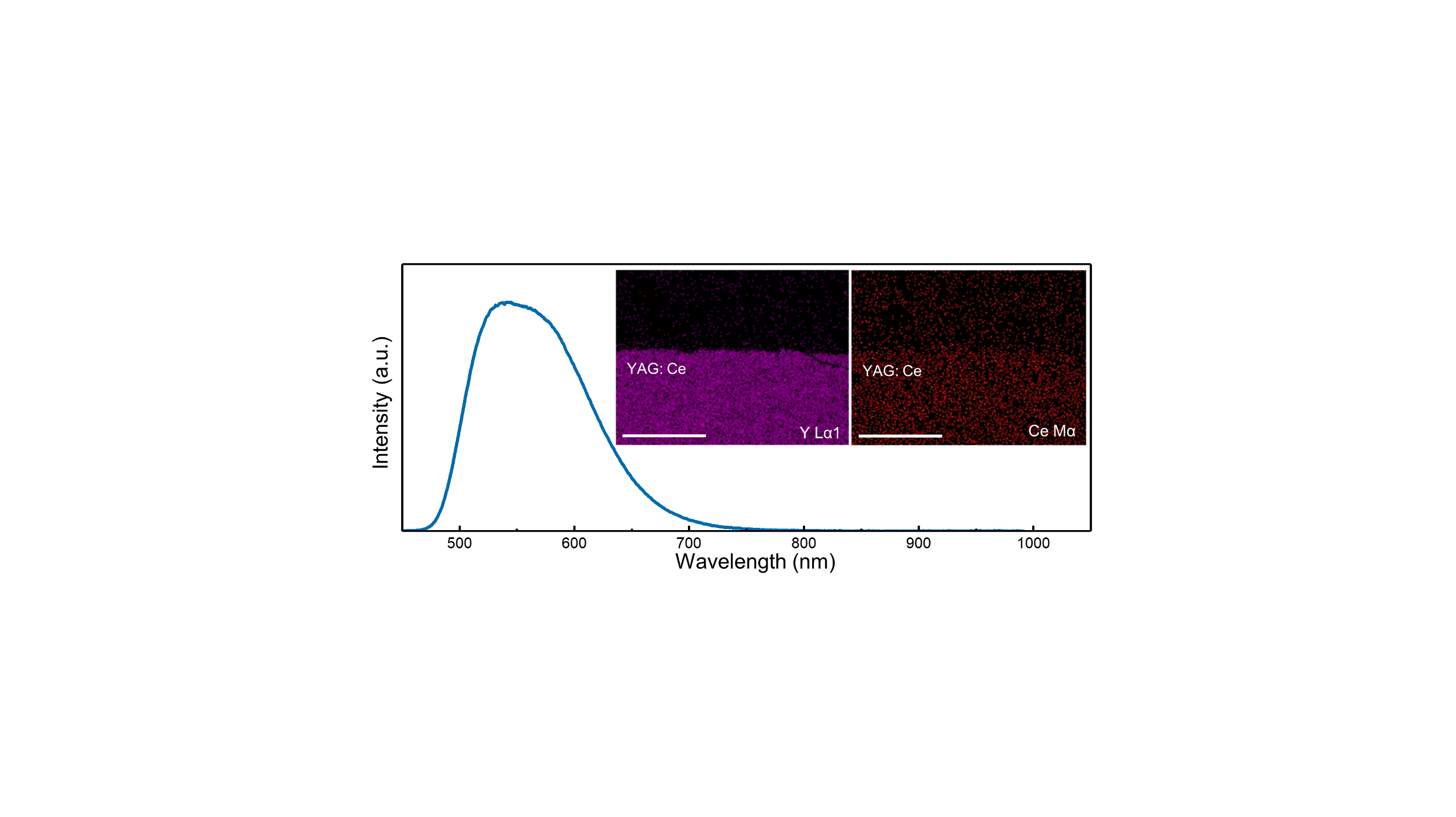


**Supplementary Note Fig. 2.** The emission spectrum of Ce: YAG and SEM element mapping image of Y and Ce (illustration).

**Supplementary Note 3：Decoder training method**

In order to reduce the requirement for massive data set and long training process, a known sample (hole with R ~5 μm) is introduced as a probe to check the imaging process. First, a standard 5 μm circular hole is used as the sample. By using the original scintillator without the metasurface, the saturated long exposure image is gained as the ground truth of the sample. By default, only the data within the edge of the small hole (light-transmitting part) of the long-exposure image is valid. By using the scintillator with affixing metasurface, short exposure samples are also obtained as the sample set. When the additional structure is replaced, the short exposure image must be reacquired.

We then use the simple RNN method to obtain the decoder. The extended inference about the theory of coherent light propagation shows that the rotation invariance should be guaranteed for the single zero-order central spot intensity of the hole. Therefore, deconvolution is performed to obtain the initial input sample $\chi_{\mathrm{in}}$, and the central zero-order spot is changed to the ideal hole image. This is defined as the first state of the Markov chain $\chi\left( n \right)$. Linear rectification operation ReLU and normalisation have been completed to avoid additional errors. Subsequently, since $\chi\left( n \right)$ must be hermit positive definite, its arithmetic square root matrix $\zeta\left( n \right)$ can always be defined as the amplitude transfer function of the system under coherent illumination. Next, we define $\chi\left( 2 \right)=F\left[ \zeta\left( 1 \right) \right]$ to complete the transmission of information to $\chi\left( n \right)$. According to the above analysis, $F\left( \zeta\right)$ includes the following operations: The image set of 0-order image at each angle is averaged, and the high-order diffraction signal in $\chi_{\mathrm{in}}$ is used to replace the signal beyond zero-order in the $\zeta\left( 1 \right)$ after square. Therefore, the information propagates from the high-order diffraction signal to the low-order diffraction signal, and the training gets completed in the forward propagation until the fine balance condition is reached. The Euclid norm of the error matrix is used as the loss function of the training, and the changing trends of l_1_-norm and l_2_-norm are shown in the text.

As mentioned above, the RNN is built. Training of a typical generator takes ~0.2 h to complete with 60 epochs.

**Supplementary Note 4：Calculation of the modulation transfer function (MTF)**

In order to further verify the effective improvement of image quality by SysK_ξ3_, we use the edge method[1] to evaluate the hypotenuse in the image for calculating the MTF. As shown in Supplementary Note Fig. 3, a resolution target of 15 μm period provides a standard hypotenuse for MTF analysis. The calculated MTF of SysK_ξ3_ and Nsys shows that SysK_ξ3_ achieves effective signal enhancement in a wide range of the spatial frequency before cut-off, which also highly corresponds to the PSD result of Fig. 4c in the manuscript.


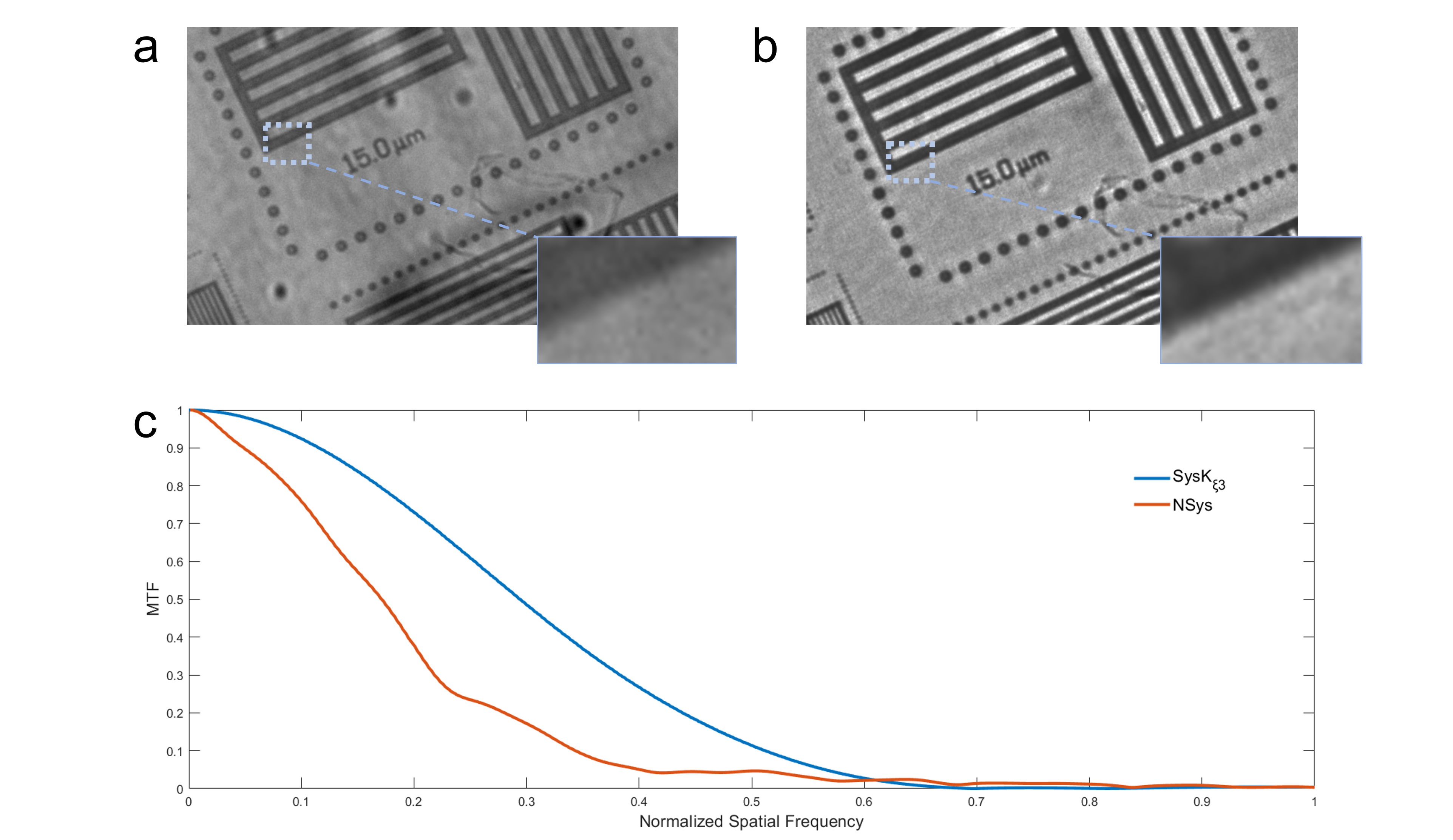


**Supplementary Note Fig. 3**. The resolution target observed by (a) SysK_ξ3_ and (b) NSys. (c) The calculated MTF of the two frameworks via edge method.

**Supplementary Note 5：Optical Information Extraction Promoted by The Fused Metasurface Imaging Method**

Our work focuses on the Ce: YAG scintillator material with a luminescence spectrum ranging from 400nm to 700nm. The refractive index of scintillator is 1.82, and the total internal reflection angle $\theta_{c}$ is calculated to be 33.3°, indicating a significant loss of approximately 90% of the photons due to internal reflection via the equation below,

$$\eta=\frac{1}{2}\int_{0}^{\theta_{c}} \sin\theta d\theta$$

Supplementary Note Fig. 4a clearly demonstrates the superior energy extraction efficiency provided by the proposed optical encoder compared to the unencoded region of the scintillator in a single capture process. It is evident that the proposed optical encoder provides a remarkable enhancement in photon extraction efficiency. The intensity cut-off values of the dashed lines (Supplementary Note Fig. 4b) reveal that our selected $SysK_{\xi3}$ encoder achieves approximately a 2.2-fold improvement in energy extraction efficiency compared to traditional methods. Additionally, the emission spectra of the visible wavelength range from the normal direction (Supplementary Note Fig. 4c) further support this conclusion.


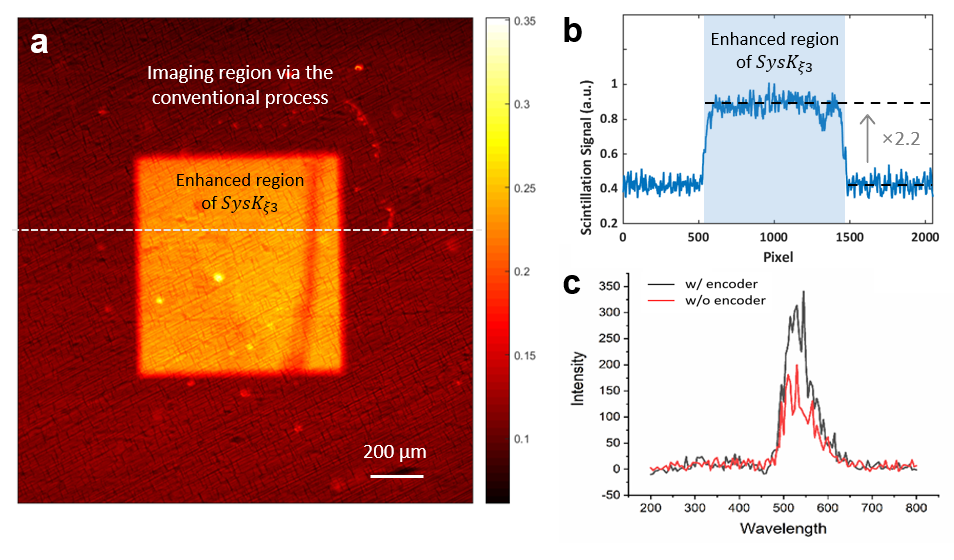


**Supplementary Note Fig. 4**. Measurement of proposed framework comparing to the conventional process. (a) Imaging regions via the conventional process and the proposed $SysK_{\xi3}$ encoder in a single capture. (b) The intensity comparison at the indicated position highlighted by the dashed lines in (a). (c) The emitted spectra in the normal direction of the scintillator in the visible range.

**Supplementary Note 6：Non-reference Quality Assessment of CT Reconstructed Images**

For evaluating the quality of three-dimensional CT reconstructed images, we utilized widely adopted non-reference quality assessment methods, namely the Perception-based Image Quality Evaluator (PIQE) and Natural Image Quality Assessment Method (NIQE)[2, 3]. As the smaller scores indicate the better perceived quality of the images, the calculations using PIQE and NIQE corroborated the improvement in image quality. Supplementary Note Table 1 shows the PIQE and NIQE results of the reconstructed vertebra and rib slices via $SysK_{\xi3}$and NSys, respectively.

**Supplementary Note Table 1.** Performance Comparison between the Proposed Fused Metasurface Imaging and Conventional Process

|  | **Rib** | | **Vertebra** | |
| --- | --- | --- | --- | --- |
|  | PIQE (a.u.) | NIQE (a.u.) | PIQE (a.u.) | NIQE (a.u.) |
| NSys | 82.702 | 15.394 | 68.417 | 20.410 |
| $SysK_{\xi3}$ | 69.329 | 10.524 | 65.162 | 19.229 |


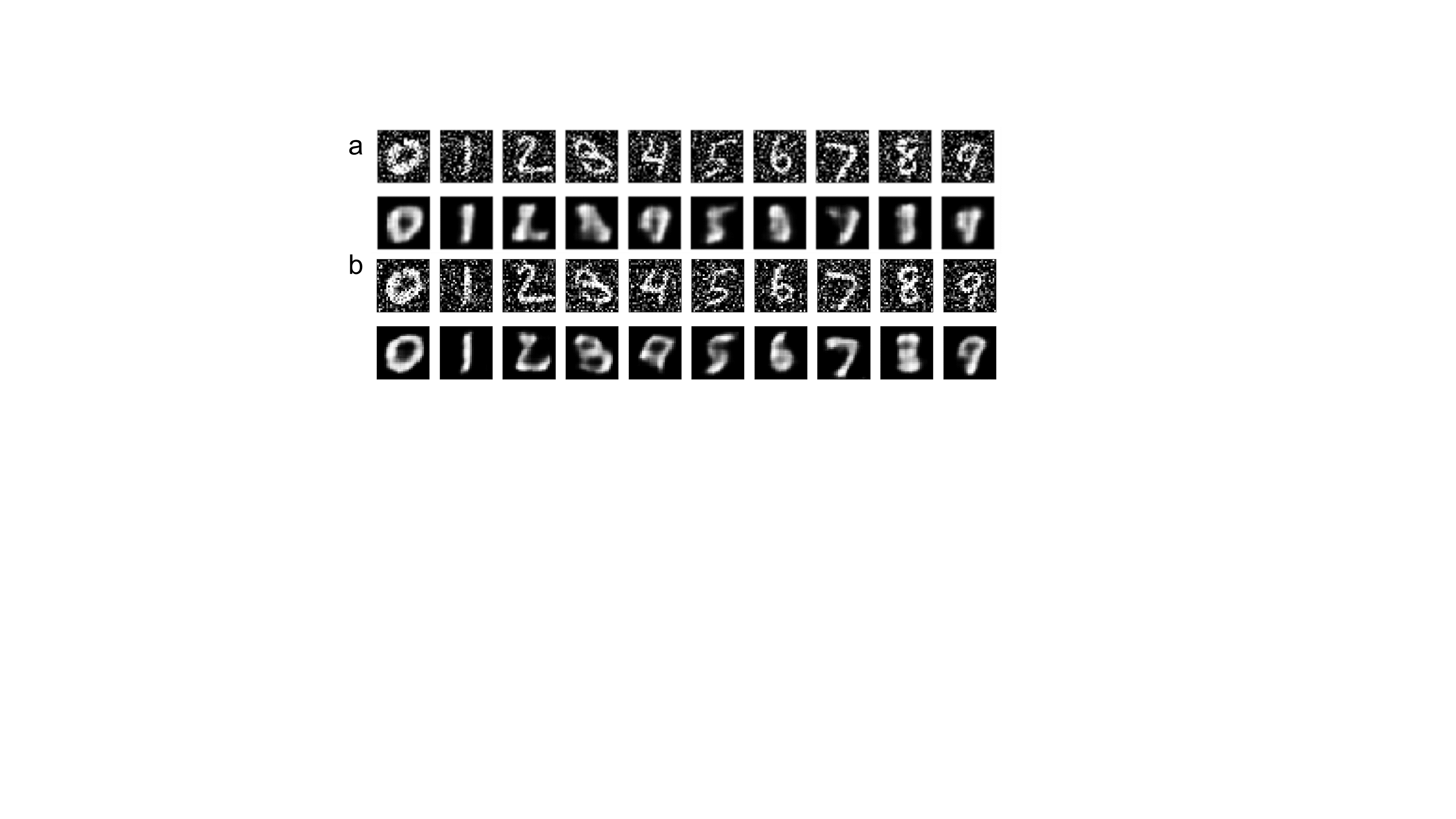


**Fig. 1.** The images (top column) generated by classic electronic CAE network with the typical 200 epochs training (bottom column).


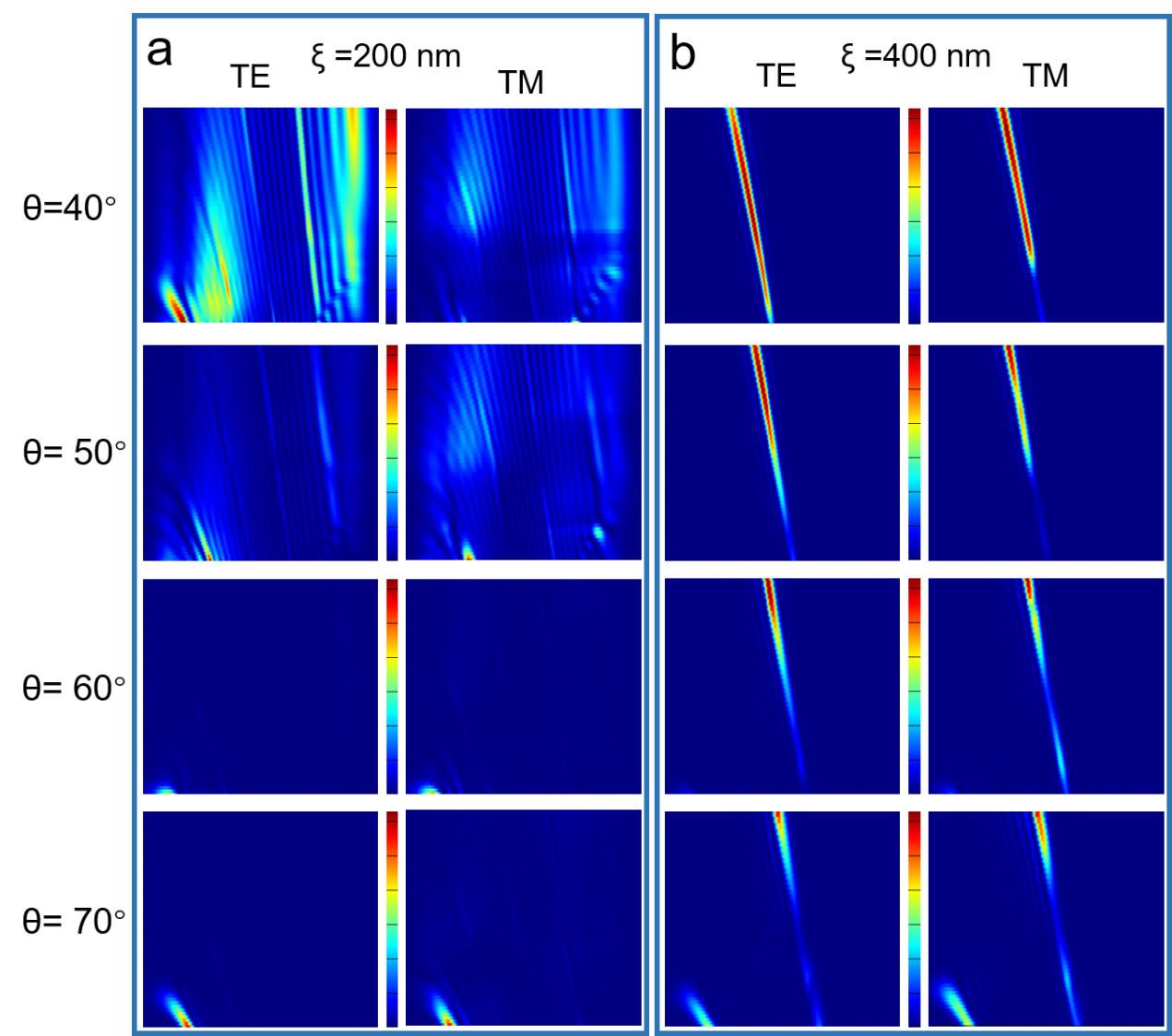


**Fig. 2.** Simulation design strategy of the convolutional kernel for texture extraction. (a) The diffraction patterns of the metasurface with a 200 nm period, (b) and 400 nm period, with each incident angle θ. The longitudinal axis is the wavelength, and the transverse axis is the exit angle (the centre is 0°) for each image. The colour scale is independent for each image.


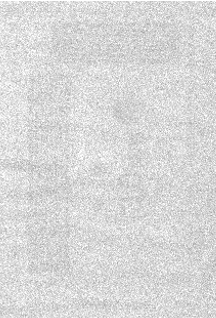


**Fig. 3.** The feature vectors Ŝ of periodic structural sample collected by the camera.


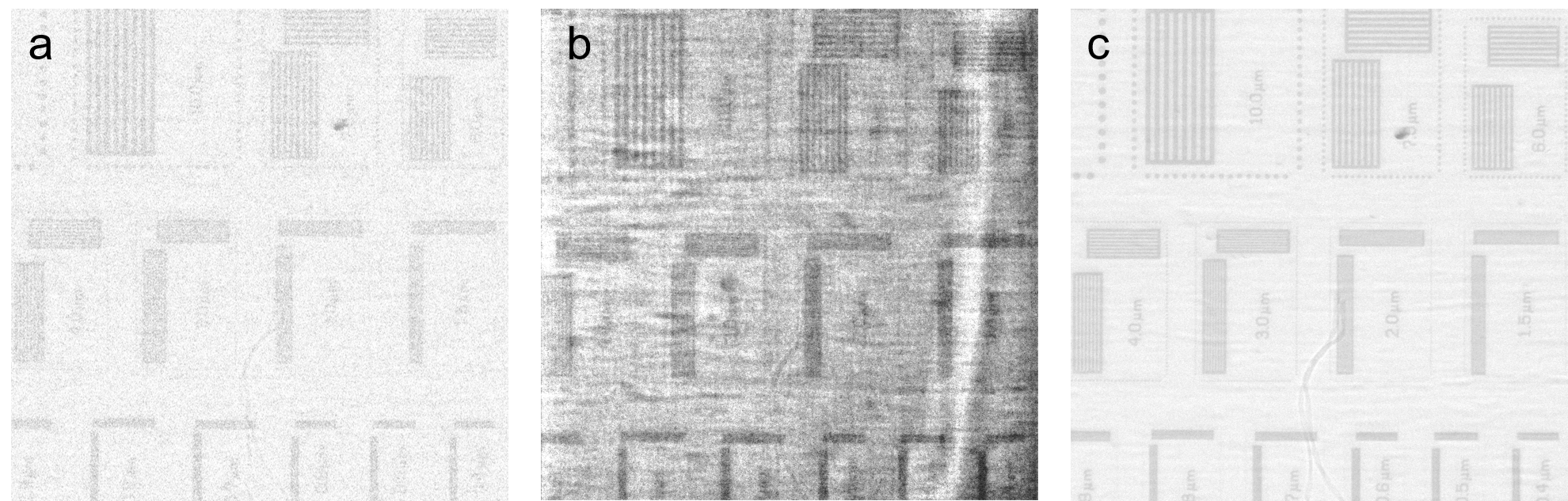


**Fig. 4.** The large-area images corresponding to SNR curve which (a) observed directly, (b) generated from SysK_ξ3_, and (c) LEI.


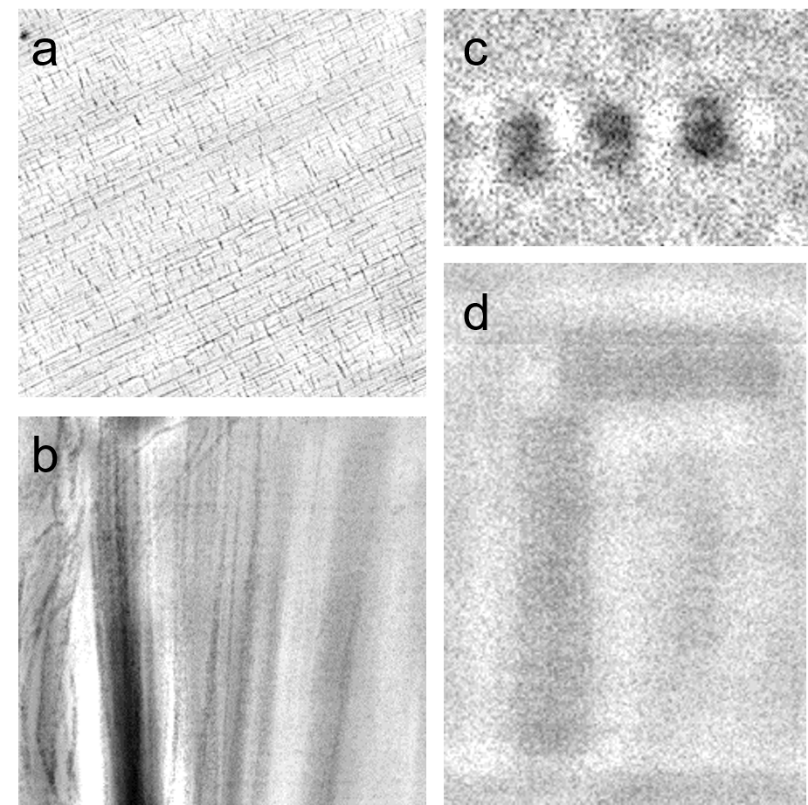


**Fig. 5.** The images of (a) the straw tissue, (b) fins, (c) the number “239” etched on the SiN_x_ film, and (d) the periodic structural sample generated by the NSys with decoder by the same algorithm.


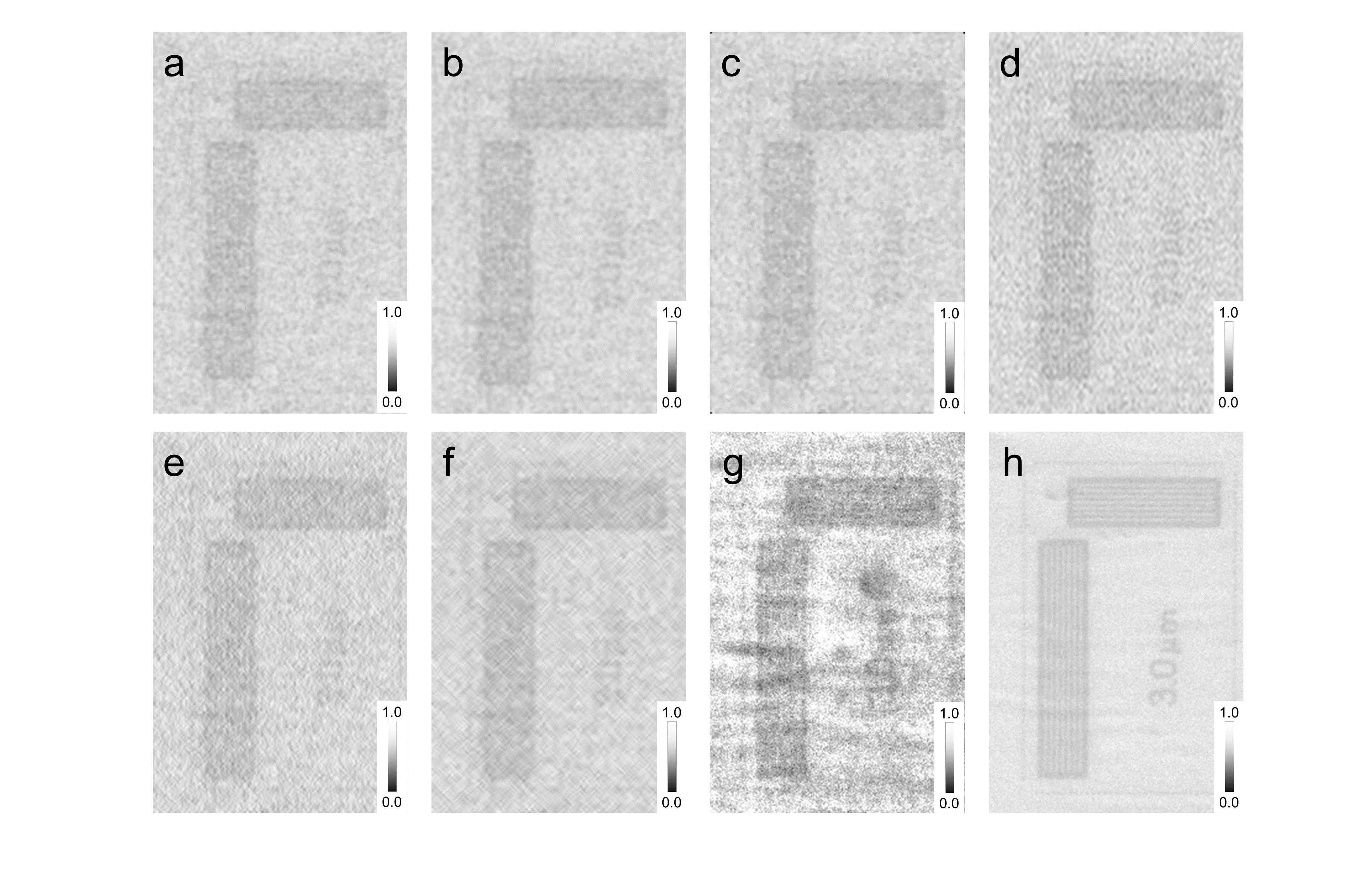


**Fig. 6.** The images of periodic structural sample generated by (a) Gaussian filter, (b) mean filter, (c) median filter, (d)low-pass filter, and (e-f) two kind of wavelet filters. (g) The image generated fromSysK_ξ3_ and corresponding (h) LEI are used for providing direct comparison.


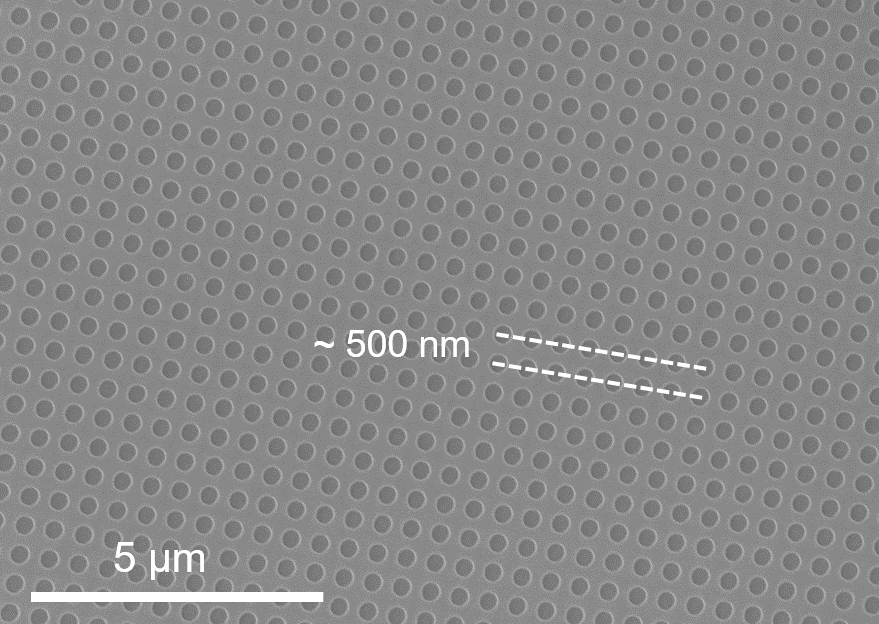


**Fig. 7.** The SEM image of the metasurface with ***ξ*** = 500 nm (K_ξ5_).


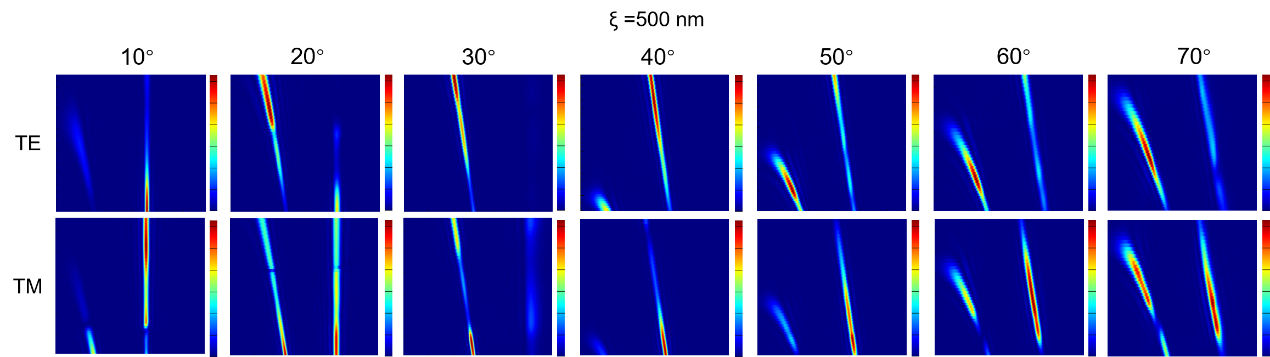


**Fig. 8.** The diffraction patterns of the metasurface with 500 nm period, in each incident angle, respectively. The longitudinal axis is the fluorescence wavelength, and the transverse axis is the exit angle (the center is 0°) for each image. The color scale is independent for each image.


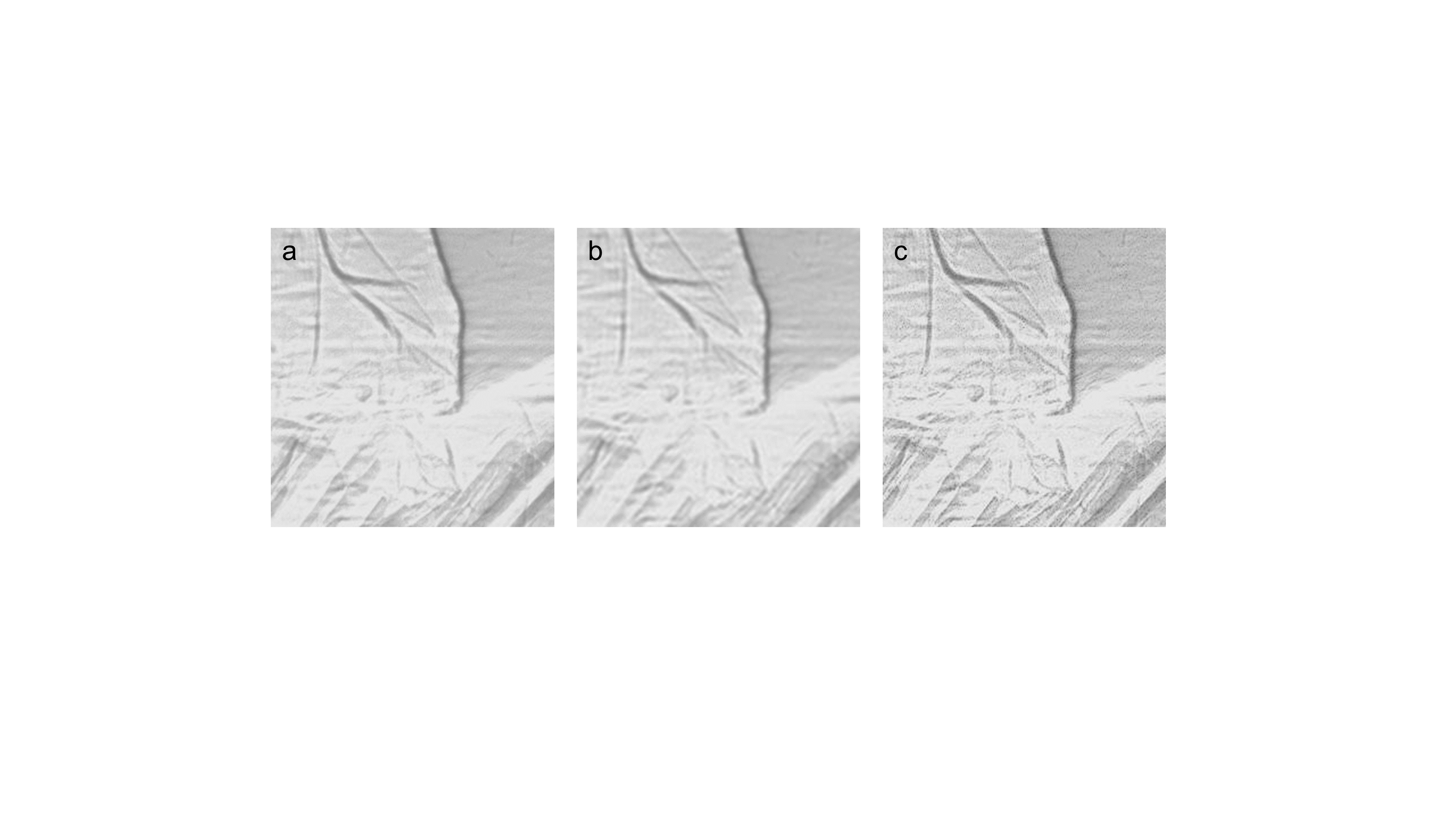


**Fig. 9.** (a) The original image. (b) The image using low-pass filtering. (c) The image using Laplacian sharpening.


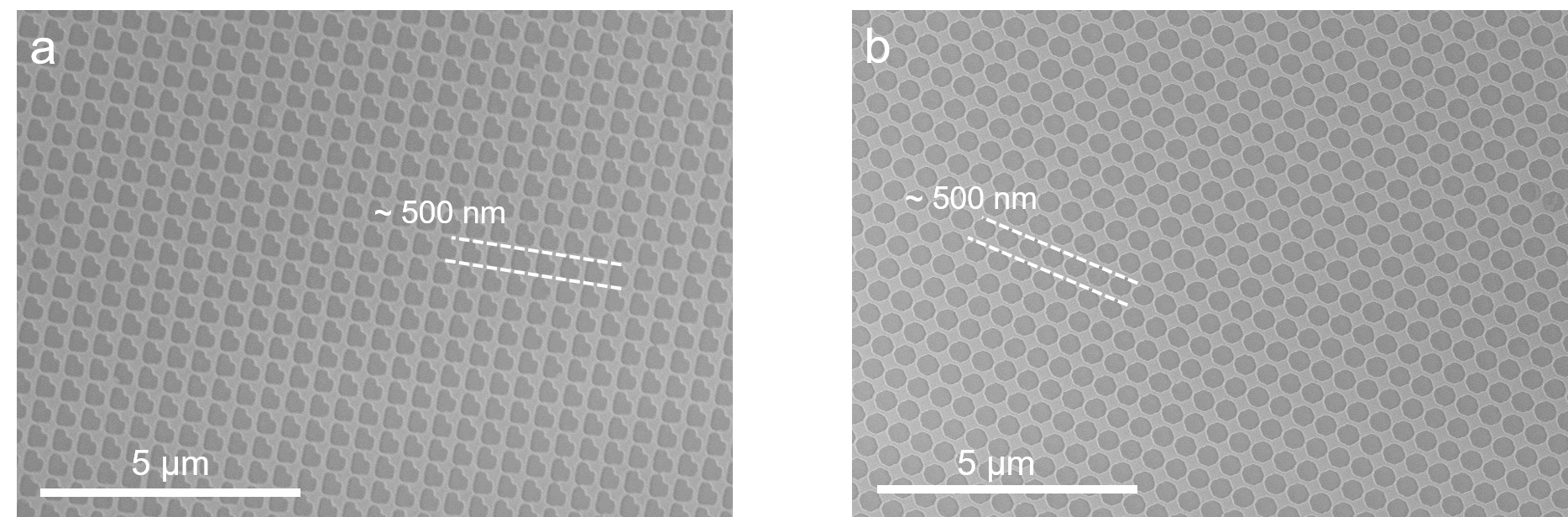


**Fig. 10.** The SEM image of the (a) octagon metasurface (K_octagon_), and (b) L-shaped metasurface (K_L-shaped_).


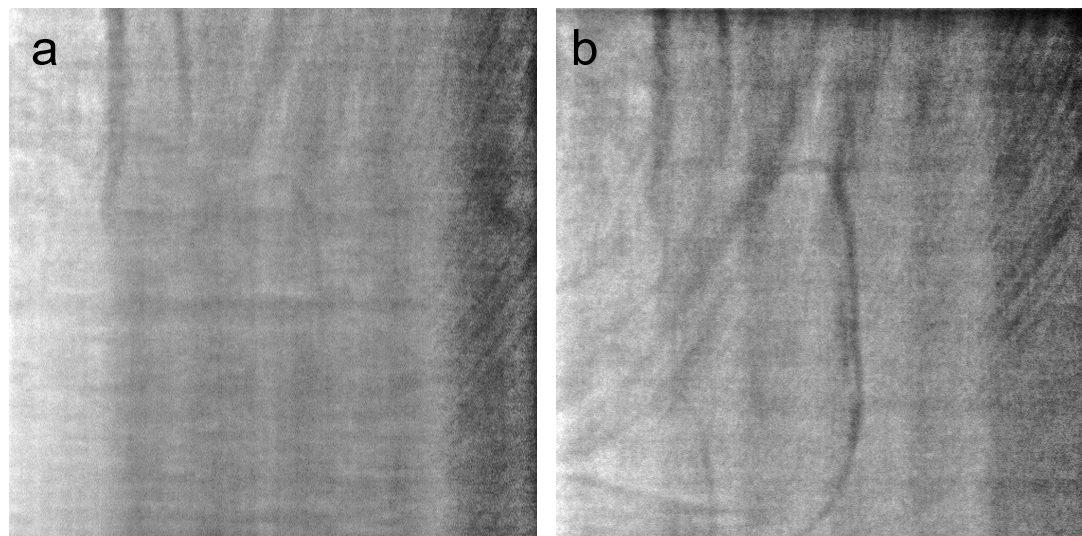


**Fig. 11.** Images of the compound eye region of the moth observed by the framework with (a) K_octagon_, and (b) K_L-shaped_.

## Reference

1. P. B. Greer, and T. van Doorn, "Evaluation of an algorithm for the assessment of the MTF using an edge method," Medical physics **27 9**, 2048-2059 (2000).

2. N. Venkatanath, D. Praneeth, B. Maruthi Chandrasekhar, S. S. Channappayya, and S. S. Medasani, "Blind image quality evaluation using perception based features," in *2015 Twenty First National Conference on Communications (NCC)*(2015), pp. 1-6.

3. A. Mittal, R. Soundararajan, and A. C. Bovik, "Making a “Completely Blind” Image Quality Analyzer," IEEE Signal Processing Letters **20**, 209-212 (2013).
